# From Ornamental Value to Antioxidant Activity: Comparative Phytochemistry of *Lavandula* Species and Cultivars

**DOI:** 10.3390/metabo16010034

**Published:** 2025-12-30

**Authors:** Andrea Baptista, Cecilia Brunetti, Dalila Pasquini, Luana Beatriz dos Santos Nascimento, Cassandra Detti, Francesco Ferrini, Sara Beltrami, Antonella Gori

**Affiliations:** 1 Institute for Sustainable Plant Protection, National Research Council of Italy (CNR), Via Madonna del Piano 10, Sesto Fiorentino, I-50019 Florence, Italy; andreacarolinabaptistaruiz@cnr.it (A.B.); francesco.ferrini@unifi.it (F.F.); antonella.gori@unifi.it (A.G.); 2 Department of Agriculture, Food, Environment and Forestry, University of Florence, Viale delle Idee 30, Sesto Fiorentino, I-50019 Florence, Italy; dalila.pasquini@scionresearch.com (D.P.); luanabeatriz.dossantosnascimento@unifi.it (L.B.d.S.N.); cassandra.detti@unifi.it (C.D.); sara.beltrami@unifi.it (S.B.); 3 Bioeconomy Science Institute, Titokorangi Drive, Private Bag 3020, Rotorua 3046, New Zealand

**Keywords:** antioxidant activity, *Lavandula* species, phenolic compounds, terpenes

## Abstract

**Background:** *Lavandula* (Lamiaceae) includes numerous species, cultivars, and hybrids widely cultivated for both their ornamental traits and for functional uses in perfumery, nutrition, medicinal, and cosmetic applications. **Objectives:** This study characterized the phytochemical profiles of three species (*Lavandula stoechas* L., *Lavandula latifolia* Medik., and *Lavandula angustifolia* Mill.), two cultivars (*L. stoechas* ‘Alba’ L. and *L. angustifolia* ‘Krajova’ Mill.), and the interspecific hybrid *Lavandula* × *intermedia* ‘Alba’ Emeric ex Loisel. **Methods:** All grown species and cultivars were maintained under uniform environmental and harvested simultaneously, to provide a comparative assessment of their terpene and polyphenol profiles and content, as well as their associated antioxidant activity. **Results:** HPLC-DAD/QTOF-MS analysis revealed differences in flavonoid and hydroxycinnamic acid content among species and cultivars. The main compounds identified were glycosylated derivatives of coumaric, caffeic, and ferulic acids, along with luteolin and apigenin derivatives. *L. latifolia* Medik. exhibited the highest hydroxycinnamic acid content (5.306 ± 1.265 mg/g FW), whereas *L. stoechas* ‘Alba’ L. showed the highest flavonoid concentration (2.537 ± 0.192 mg/g FW). GC-MS analysis indicated that hydrocarbon and oxygenated monoterpenes were the predominant terpene classes, with the highest levels recorded in *L. stoechas* L. (1922.09 ± 144.12 ng/g FW oxygenated; 945.89 ± 159.26 ng/g FW hydrocarbon monoterpenes). Antioxidant activity, assessed via DPPH and FRAP assays, was significantly correlated with flavonoid content across species, cultivars, and the hybrid. **Conclusions:** Intraspecific and interspecific variability within the *Lavandula* genus influences antioxidant activity and determines its suitability for different applications.

## 1. Introduction

The *Lavandula* genus (Lamiaceae) includes numerous herbaceous, annual, or perennial shrub species characterized by a typical spike inflorescence. Members of this genus are aromatic plants widely cultivated throughout the world, appreciated both for their ornamental value and their medicinal applications. These properties are mainly linked to their high content of essential oils and bioactive compounds, which confer both organoleptic and biological activities. Scientific and practical interest in *Lavandula* metabolites is continually growing, particularly due to their ability to counteract oxidative stress, a property widely exploited in the cosmetic and food sectors [1,2,3].

Among the main metabolites present in *Lavandula* species, there are terpenes (both monoterpenes and sesquiterpenes) and polyphenols (in particular flavonoids and phenolic acids) [4]. The most abundant terpenes include linalool, linalyl acetate, camphor, limonene, and cineole [5]. These molecules are known for their antioxidant, antimicrobial, and anti-inflammatory activities and are widely used in the cosmetic, pharmaceutical, food, and aromatherapy sectors [6,7,8]. Among the terpene class, linalool, the main component of *Lavandula* essential oil, has demonstrated antioxidant, antiplatelet, and inhibitory effects on acetylcholinesterase, suggesting its potential use as a therapeutic agent in neurodegenerative diseases such as Alzheimer’s [9]. Additionally, lavender essential oils characterized by a high content of 1,8-cineole, such as those of the species *Latifolia*, have exhibited efficacy as analgesics. Their inhalation has been reported to relieve pain and reduce hair loss and anxiety in patients undergoing chemotherapy [10], thus making aromatherapy with lavender oils a valid complementary palliative therapy for mood improvement [11].

Polyphenols represent a further major class of natural antioxidants with potential nutraceutical applications. Leaf flavonoids in the *Lavandula*, besides constituting taxonomic markers, are also the main target of green extraction methodologies aimed at obtaining polyphenol-enriched extracts [7,12]. These extracts find application in cosmetics (e.g., anti-aging creams and lotions) and functional foods, enhancing their nutraceutical value. Furthermore, the evaluation of antioxidant activity may represent a useful functional indicator to screen the potential application of different plant species. Indeed, it has been observed that extracts of different *Lavandula* species exhibit variable antioxidant capacities, primarily due to the presence and/or varying abundance of specific terpenes and polyphenols responsible for the observed biological activity [13]. For example, Radi et al. [14] highlighted that *Lavandula stoechas* L. possesses greater antioxidant activity than *Lavandula abrialis*, likely due to the fact that L. *stoechas* L. presents a higher content of cubenol, fenchone, and camphor, whereas *L. abrialis* is mainly characterized by the presence of linalool, linalyl acetate, and camphor. Antibacterial activity was also found to be closely related to the chemical composition of the *Lavandula* species examined. Different geographical origins, as well as intraspecific variability among cultivars, strongly influence the antibacterial potential, both in terms of the intensity and spectrum of activity toward specific microorganisms [15]. This evidence emphasizes the importance of a careful phytochemical characterization of *Lavandula* species and cultivars, not only to guide their functional applications but also to support their ornamental selection and cultivation.

Although several *Lavandula* species and cultivars have been previously studied, comparative analyses conducted under uniform cultivation and harvest conditions remain scarce. This limits the reliability of cross-study comparisons, given the strong environmental influence on secondary metabolites and bioactivity. In addition, the relationship between polyphenol composition and antioxidant capacity across *Lavandula* species, cultivars, and hybrids is still insufficiently understood, despite their potential for targeted applications. Intra- and interspecific variability in secondary metabolites could also be useful from an ornamental perspective, as chemical diversity may contribute to differences in aroma, tolerance to biotic and abiotic stresses, and, thus, horticultural performance.

The present study provides a comparative assessment of bioactive compounds and antioxidant activity of three lavender species (*L. stoechas* L., *L. latifolia* Medik., and *L. angustifolia* Mill.), two cultivars (*L. stoechas* ‘Alba’ L. and *L. angustifolia* ‘Krajova’ Mill.), and the sterile hybrid *Lavandula* × *intermedia* ‘Alba’ Emeric ex Loisel. All these species and cultivars are widely valued for their ornamental value due to their strong aesthetic appeal, ease of growth, and tolerance to adverse environmental conditions. Additionally, these species and cultivars possess notable bioactive properties, as their elevated polyphenol and terpene contents make them promising candidates for applications in the medical and cosmetic industries [16]. All adult plants were cultivated under the same environmental conditions (temperature, irrigation, and natural light) and harvested simultaneously, allowing a more consistent comparison of their phytochemical profiles (focusing on terpenes and polyphenols) and antioxidant activities.

Specifically, this study focuses on the following objectives: (i) to identify qualitative and quantitative differences in polyphenols and terpenes among the different genotypes, and (ii) to evaluate possible correlations between polyphenolic compounds and antioxidant activity to identify chemical markers useful for both functional and ornamental valorization.

## 2. Materials and Methods

### 2.1. Reagents

Methanol (HPLC-grade) ≥ 99.9% purity, Sigma-Aldrich (Darmstadt, Germany); ethanol (HPLC-grade) ≥ 99.9%, Sigma-Aldrich (Darmstadt, Germany); water for chromatography (LC-MS Grade), LiChrosolv^®^, Sigma-Aldrich (Darmstadt, Germany); formic acid 98–100%, for LC-MS LiChropur, Supelco (Bellefonte, PA, USA). The standards and standard mixes were supplied by Sigma-Aldrich (Darmstadt, Germany).

### 2.2. Plant Material Sampling

The three lavender species analyzed (*L. stoechas* L., *L. latifolia* Medik., and *L. angustifolia* Mill.) are primarily found in the Mediterranean region and are commonly known as French lavender, spike lavender, and English lavender, respectively [17,18,19]. Meanwhile, the hybrid species *Lavandula* × *intermedia* Emeric ex Loisel is commonly referred to as lavandin [19], while the *L. angustifolia* cultivar ‘Krajova’ Mill., originating from Central Europe, particularly from the Czech Republic and Slovakia, is known as Czech Lavandula [20]. Furthermore, *L*. *stoechas* ‘Alba’ L. is a cultivar of *L. stoechas* L., commonly known as White Spanish Lavender.

Five-year-old plants of the aforementioned *Lavandula* species, cultivars, and the hybrid were grown in the Green Economy and Agriculture (GEA) Park located in Pistoia, Italy (43°55′9.077″ N, 10°54′25.913″ E). The plants were cultivated outdoors in pots filled with commercial soil, kept well-irrigated, and exposed to natural sunlight. All cultivars were grown under identical conditions; ten plants per cultivar/species were used for leaf sampling, and three replicates (*n* = 3) were analyzed. Leaves were sampled on 27 January 2021, from 10:00 to 11:00 a.m., from six branches between 30 and 40 cm in length, then immediately frozen in liquid nitrogen and stored at −80 °C until analysis.

### 2.3. Extraction and Analysis of Phenolic Compounds

For the analysis of phenolic compounds, approximately 70 mg of *Lavandula* leaf samples were extracted with 3 × 1.8 mL of ethanol:water (75:25%, *v*/*v*) at pH 2.5, adjusted with formic acid. Extraction was performed using Ultrasound-Assisted Extraction in an ultrasonic bath (BioClass^®^ CP104, 39 kHz, 100 W, CEIA S.p.A., Arezzo, Italy) at a low temperature (approximately 4–6 °C) for 30 min. The samples were then centrifuged at 9000 rpm for 5 min at 5 °C (ALC^®^ 4239R, Milan, Italy). The resulting supernatants were defatted using *n*-hexane (3 × 1.8 mL) to remove lipophilic compounds. The ethanol extracts were subsequently evaporated to dryness using a speed-vac concentrator (Eppendorf^®^ Concentrator Plus, Hamburg, Germany) for 2 h. Finally, the dried extracts were resuspended in 200 μL of methanol: water (50:50, *v*/*v*; pH 2.5, adjusted with formic acid) and analyzed by HPLC-DAD and HPLC-ESI/Q-TOF.

#### 2.3.1. HPLC-DAD Analysis

Aliquots (10 μL) of the samples were injected into a Perkin^®^ Elmer Flexar liquid chromatograph equipped with a quaternary 200Q/410 pump and an LC 200 diode array detector (DAD) (all from Perkin Elmer^®^, Bradford, CT, USA). The mobile phase consisted of a gradient of (A) acidified water (at pH 2.5, adjusted with formic acid) and (B) acetonitrile (at pH 2.5, adjusted with formic acid), while the stationary phase consisted of an Agilent^®^Zorbax^®^ SB-C18 column (250 × 4.6 mm, 5 μm), kept at 30 °C. This analytical method is consistent with those previously reported in the scientific literature [21]. Briefly, the following gradient was applied: 10 min re-equilibration (3% B), 0–1 min 3% B, then 1–55 min (3–40% B), 55–60 min (40% B), 60–61 min (40–3% B), 61–62 min (3% B); the flow elution was 0.6 mL min^−1^ [22]. For the identification and quantification, chromatograms were recorded at wavelengths of 280, 330, and 350 nm, and the peak area at the wavelength corresponding to the maximum absorption of each compound class was selected for the quantification. The preliminary identification of the predominant phenolic compounds was carried out by thorough examination of their UV spectra and then confirmed by HPLC-MS analysis (see Section 2.3.2). Quantification was then performed using five concentration point calibration curves of the following reference standards: apigenin-7-*O*-glucoside, luteolin-7-*O*-glucoside, ferulic acid, coumaric acid, and rosmarinic acid. The quantitative data for phenolic compounds are presented in the main text as the total amounts of hydroxycinnamic acid derivatives and flavonoids. Results are reported on a fresh weight (FW) basis.

#### 2.3.2. HPLC-ESI/Q-TOF-MS Analysis

To identify the phenolic compounds, a 1 µL aliquot of the *Lavandula* extracts was injected into an Agilent^®^ 6530 QTOF mass spectrometer equipped with a quadrupole-time-of-flight (QTOF) analyzer, operating in negative electrospray ionization (ESI^−^) mode and coupled to a diode array detector (DAD). The method was adapted from that used in [23]. Chromatographic separation was carried out on an InfinityLab Poroshell 120 EC-C18 column (2.1 × 50 mm, 2.7 µm) equipped with a Poroshell 120 SB-C18 UHPLC guard column (2.1 × 5 mm, 2.7 µm). The mobile phase consisted of solvent A (water with 0.1% formic acid) and solvent B (acetonitrile with 0.1% formic acid), using the following gradient program: 0–5 min, 3% B; 5–25 min, 3–20% B; 25–30 min, 20% B; 30–50 min, 20–40% B; 50–70 min, 40–97% B; 70–73 min, 97% B; and 74–82 min, 97–3% B, for re-equilibration. The flow rate was set to 0.3 mL min^−1^. The applied ESI parameters were set as follows: capillary voltage, 4000 V; fragmentor 115 V; skimmer 60 V; Oct 1 RF Vpp 750 V; pressure of nebulizer 20 psi; drying gas temperature 325 ◦C; sheath gas temperature 400 °C. Identification was performed based on accurate mass determination, the detection of the deprotonated molecular ion in negative mode ([M–H]^−^), and comparison with data reported in the literature.

#### 2.3.3. Antioxidant Activity Assays

##### FRAP (Ferric-Reducing Antioxidant Power) Assay

To investigate antioxidant activity in terms of ferrous ion reduction, the FRAP (ferric-reducing antioxidant power) assay was carried out. This method is based on the reduction of the ferric 2,4,6-tris(2-pyridyl)-1,3,5-triazine (Fe^3+^–TPTZ) complex to the Fe^2+^–TPTZ complex at acidic pH, resulting in a color change of the solution to an intense blue. The ferric-reducing antioxidant power (FRAP) assay was carried out using a commercial kit (Sigma-Aldrich, Darmstadt, Germany). The procedure follows the method reported in [21] with slight modifications. Briefly, 10 μL of the ethanolic extracts were mixed with 152 μL of buffer, 19 μL of FeCl_3_ solution, and 19 μL of FRAP reagent. Control blanks contained the same extract solvents but without the FRAP reagent. After incubation for 60 min at 37 °C, absorbance was recorded at 594 nm using a SpectraMax^®^ microplate reader (Molecular Devices, San Jose, CA, USA). Calibration curves were generated using Fe(II) (FeSO_4_·7H_2_O) standards ranging from 4 to 20 nmol. The assay was performed in triplicate, and the calibration plot showed a good linear fit. The test results are expressed as millimolar (mM) ferrous equivalents.

##### DPPH (2,2-Diphenyl-1-Picrylhydrazyl) Radical-Scavenging Activity Assay

Antioxidant activity was determined using the DPPH assay by mixing 500 µL of diluted extracts (10 µL of extract in 5 mL of ethanol) with 500 µL of a commercial DPPH solution (0.1 mM in methanol; Sigma-Aldrich, Merck KGaA, Darmstadt, Germany) using the method reported in [24] with slight modifications. The mixture was incubated in the dark at room temperature for 45 min, and absorbance was recorded at 518 nm using a PerkinElmer^®^ UV/Vis spectrophotometer (Lambda 25, PerkinElmer, Bradford, CT, USA). Absorbance was measured for the blank (500 µL of methanol + 500 µL of methanol), the negative control (500 µL of the DPPH solution), and the positive control (rosmarinic acid). The EC_50_ (effective concentration at 50%) values were calculated by linear regression and follow the protocol described in [24], whereas the percentage of antioxidant activity was determined using the following equation:

AA% = 100 − {[(ABS_sample_ − ABS_blank_) × 100]/ABS_negative control_}

### 2.4. Terpene Extraction and Analysis

For the extraction, 0.5 g of leaf tissue was placed in a 2 mL glass vial with 1 mL of *n*-heptane. The mixture underwent three 15 min sonication cycles, followed by agitation for 24 h at a constant temperature of 35 °C. After centrifugation at 4000 rpm for 10 min, a 100 μL aliquot of the supernatant was collected for GC–MS analysis (for additional details, refer to [22]). Ten plants were used as the source of material, with three replicates per species/cultivar. Analysis was performed on an Agilent 7820A gas chromatograph coupled to a 5977E single quadrupole mass selective detector (Agilent Technologies, Palo Alto, CA, USA). A 1 μL sample was injected in splitless mode using a split/splitless injector and a Gerstel MPS2 XL autosampler (Gerstel, Mülheim an der Ruhr, Germany) equipped with a liquid injection module. Helium was used as the carrier gas at 33 psi and a flow rate of 1.2 mL min^−1^.

The separation was carried out on an Agilent DB-Wax UI capillary column (60 m × 0.25 mm, 0.5 μm film thickness). The oven temperature program was as follows: initial hold at 40 °C for 1 min, followed by a ramp of 5 °C min^−1^ to 200 °C, then 10 °C min^−1^ to 240 °C, with a final hold of 6 min. The mass spectrometer operated in electron ionization mode (70 eV), scanning in the *m*/*z* range of 40–350 at a scan rate of 3 scans per second. The data was processed using Agilent MassHunter Workstation (Qualitative Analysis-Version B.06.00 and Quantitative Analysis Version B.07.01/Build 7.1.524.0 Compound identification was verified using authentic standards and by comparing their mass spectra and retention times with those available in the NIST 11 spectral database (National Institute of Standards and Technology, Gaithersburg, MD, USA). For quantification during the reprocessing method, the chromatograms were filtered using the EIC at *m*/*z* 93 to maximize analytical selectivity and reduce matrix interferences. Quantification was performed using commercial solution standards (Terpenes Mix A, Terpenes Mix B, and Terpenes Mix) from Sigma-Aldrich (Darmstadt, Germany). Five-point calibration curves, prepared over the concentration range of 1 ppm–166 ppm, exhibited excellent linearity with R^2^ > 0.99. Results are reported as mean values of three replicates on a fresh weight (FW) basis.

### 2.5. Statistical Analysis

Statistical analysis started with an assessment of data normality using the Kolmogorov–Smirnov test. For data sets following a normal distribution, one-way ANOVA was performed. In cases where the data did not meet normality assumptions, the non-parametric Kruskal–Wallis test was applied. Post hoc comparisons between groups were conducted using both Duncan’s and Tukey’s tests. Statistical significance was considered at *p* < 0.05. Additionally, principal component analysis (PCA) was conducted to identify the terpenes and phenolic compounds that contributed most to the variation among *Lavandula* species and cultivars. Spearman correlation analysis was also carried out to assess the relationship between phenolic content and antioxidant activity. All statistical analyses were performed using SPSS Statistics, version 29.0.2.0 [25].

## 3. Results

### 3.1. Phenolic Compounds

**Figure 1:**
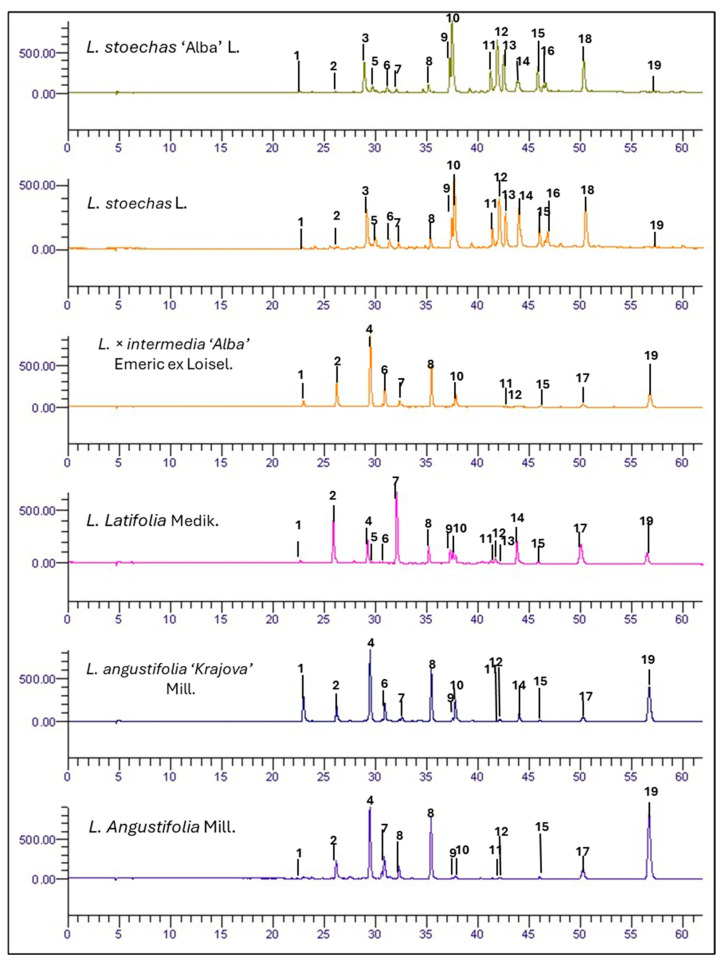
HPLC-DAD chromatograms of phenolic compounds at 330 nm for *L. stoechas* L., *L. latifolia*, *L. angustifolia* Mill., *L. × intermedia* ‘Alba’ Emeric ex Loisel, *L. stoechas* ‘Alba’ L., and *L. angustifolia* ‘Krajova’ Mill. Identified peaks are shown in Table 1, and their concentrations are expressed in mg/g FW in Table S1.

Across all samples, 19 phenolic compounds were identified (Figure 1) and grouped into two main classes: hydroxycinnamic acid derivatives and flavonoids (Table 1).

Specifically, for hydroxycinnamic acid derivatives, eleven different compounds were identified in the phenolic extracts of the six *Lavandula* genotypes (Table 1 and Table S1). These compounds are characteristic of the Lamiaceae family, contributing to plant defense against UV radiation, herbivory, viruses, and bacteria, and they are known for their antioxidant, anti-inflammatory, antimicrobial, anti-collagenase, and anti-melanogenic effects [6,16,24,26,27,28]. Interestingly, some of these compounds appeared to be specific to certain species or cultivars included in our study. For example, feruloyl tartaric acid ([M–H]^−^ ion at 325 *m*/*z*) was identified and quantified only in *L. stoechas* L. and in the cultivar *L*. *stoechas* ‘Alba’ L., confirming the findings of multiple studies reporting the presence of this phenolic ester only in this species [16,26]. Similarly, salvianolic acid B ([M–H]^−^ ion at 717 *m*/*z*) was mainly detected in *L. stoechas* L. and *L. latifolia* Medik. (see Figure S18) [2,13,16,26,29,30]. This caffeic acid tetramer is specific to these species and is notable for its high antioxidant activity, attributed to its structure that facilitates effective radical scavenging, as well as for its anticoagulant and antithrombotic properties [31]. In contrast, the highest concentrations of the isomers *trans*-*p*-coumaric acid 4-*O*-glucoside and *o*-coumaric acid 2-*O*-glucoside ([M–H]^−^ ion at *m*/*z* 325) were found in *L. angustifolia* ‘Krajova’ Mill., *L. latifolia* Medik., and *L. × intermedia* ‘Alba’ Emeric ex Loisel. In addition, ferulic acid *O*-glucoside ([M–H]^−^ = 355 *m*/*z*) was also identified in these species. These results are consistent with those reported by [28], who observed a high concentration (171.42 µg/g DW) of coumaric acid 4-*O*-glucoside in *L. angustifolia* Mill. Furthermore, the peak 17, referred to as Unknown 1, was tentatively classified as a flavonoid based on its UV spectrum and quantified within this compound family. Peaks 18 and 19, referred to as Unknowns 2 and 3, respectively, displayed spectra consistent with ferulic acid and rosmarinic acid (Figures S20 and S21), and were therefore considered part of the hydroxycinnamic acid class. However, mass spectrometry analysis did not allow for the precise identification of these compounds.

In the *Lavandula*, ethanolic extracts were also identified, and eight flavonoids belonging to the flavone subclass were quantified, including luteolin, apigenin, and their glycosylated derivatives such as luteolin 7-*O*-glucoside and apigenin 7-*O*-glucuronide (Table 1 and Table S1). Among the *Lavandula* species in our study, the 7-*O*-monoglycoside flavones were the most abundant, as reported in the literature [12,29].

**Table 1:** Peak number, tentative identification by HPLC-ESI-Q-TOF in negative mode (see spectra in Figures S3–S21), and maximum UV absorption determined by HPLC-DAD of the hydroxycinnamic acids and flavonoids identified and quantified in *Lavandula* species (*L. stoechas* L., *L. latifolia* Medik., and *L. angustifolia* Mill.), cultivars (*L*. *stoechas* ‘Alba’ L. and *L. angustifolia ‘*Krajova’ Mill.), and the hybrid *L. × intermedia* ‘Alba’ Emeric ex Loisel. Means with the same letter are not significantly different according to Duncan’s test (*p* > 0.05).

Peak	Rt (min)	Tentative Identification	[M-H]^−^	UV Max	Ref.
**Hydroxycinnamic acid and derivatives**					
**1**	22.56	1-*o*-Caffeoylglucose	341	300, 326	[32]
**2**	26.10	*trans*-*p*-Coumaric acid 4-*O*-glucoside	325	285	[33]
**3**	28.92	Feruloyltartaric acid ^a^	325	328	[16,26]
**4**	29.27	Ferulic acid *O*-glucoside ^b^	355	280, 305	[34]
**5**	30.06	Caffeic acid acetylhexoside ^a^	387	328	[35]
**7**	32.05	*o*-Coumaric acid 2-*O*-glucoside	325	275, sh 313	[36]
**8**	35.63	Ferulic acid-*O*-glucoside	355	318–320	[37,38]
**14**	43.93	Rosmarinic acid	359	328	[26]
**16**	46.56	Salvianolic acid B ^a^	717	sh 334	[27]
**18**	50.28	Unknown 2 ^a^	501	326	-
**19**	56.94	Unknown 3	727	325	-
	Flavonoids				
**6**	31.11	Apigenin *C*-hexoside	431	334	[39]
**9**	37.22	Luteolin 7-*O*-glucuronide	461	256, 267 sh, 347	[12]
**10**	37.72	Luteolin 7-*O*-glucoside	447	256, 267 sh, 350	[12]
**11**	41.23	Apigenin-7-*O*-Glucoside	431	267, 332	[26,28]
**12**	41.85	Apigenin 7-*O*-glucuronide	445	268, 333	[26]
**13**	42.55	Methyl-luteolin-O-glucuronide	475	256, 260 sh, 346	[40]
**15**	45.82	Apigenin 7-(6″-acetylglucoside) ^a^	473	267, 334	[40]
**17**	49.45	Unknown 1 ^b^	331	280, 312	-

^a^ Identified and quantified only in *L. stoechas* L. and *L*. *stoechas* ‘Alba’ L.; ^b^ identified and quantified only in *L. latifolia* Medik., L. *× intermedia* ‘Alba’ Emeric ex Loisel, *L. angustifolia* Mill., and *L. angustifolia ‘*Krajova*’* Mill. “sh”: shoulder absorption.

Following characterization, quantification of the identified compounds was performed, showing that *L. latifolia* Medik. exhibited the highest total phenolic content (6.686 ± 1.593 mg/g FW), followed by *L. angustifolia* ‘Krajova’ Mill. (5.173 ± 1.636 mg/g FW) (Table S1).

**Figure 2:**
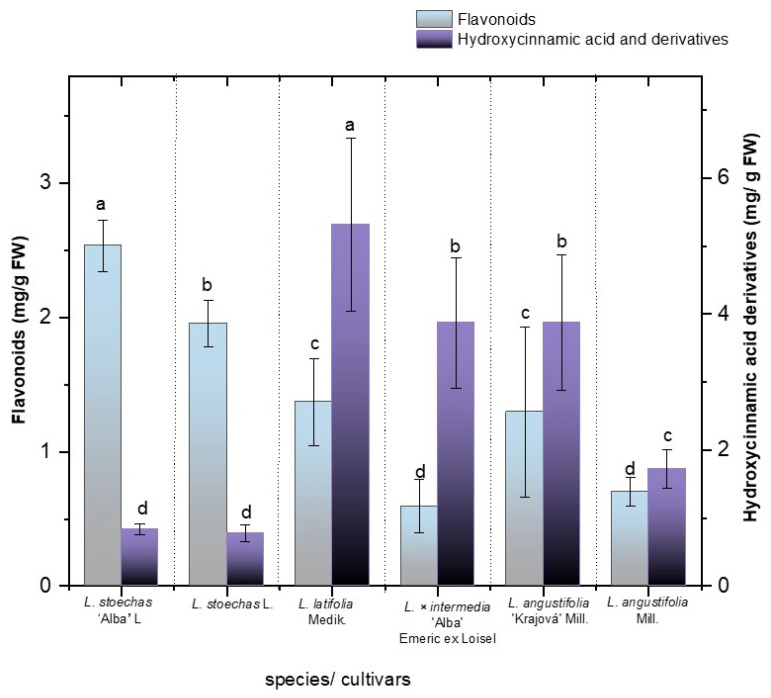
Total content of hydroxycinamic acids and flavonoids in mg/g FW in the species *L. stoechas* L., *L. latifolia* Medik., *L. angustifolia* Mill., and *L. × intermedia* ‘Alba’ Emeric ex Loisel, and the cultivars *L. stoechas* ‘Alba’ L. and *L. angustifolia* ‘Krajova’ Mill. Means with the same letter are not significantly different according to Duncan’s test (*p* > 0.05).

Among the two main phenolic classes identified, hydroxycinnamic acid derivatives (HC) and flavonoids (Figure 2), HC derivatives were the most abundant across all species and cultivars, with concentrations ranging from 0.833 to 5.306 mg/g FW, while flavonoids showed total concentrations ranging from 0.593 to 2.537 mg/g FW (Figure 2, Table S1). The analysis of hydroxycinnamic acid derivative concentrations revealed significant interspecific variation, with *L. latifolia* Medik. showing the highest levels (5.306 ± 1.265 mg/g FW), followed by *L. angustifolia* ‘Krajova’ Mill. (3.856 ± 0.280 mg/g FW) and *L. × intermedia* ‘Alba’ (3.858 ± 0.954 mg/g FW). Meanwhile, no significant difference was observed in the total HC derivatives between *L. stoechas* L. (0.777 ± 0.118 mg/g FW) and its cultivar ‘Alba’ L. (0.833 ± 0.082 mg/g FW). The amount of HC derivatives in *L. angustifolia* Mill. (1.713 ± 0.280 mg/g FW) and its cultivar *L. angustifolia* ‘Krajova’ Mill. (3.876 ± 1.002 mg/g FW) showed significant differences.

On the other hand, in terms of flavonoid concentrations, the cultivar with the highest was *L. stoechas* ‘Alba’ L. (2.537 ± 0.192 mg/g FW), followed by *L. stoechas* L. (1.956 ± 0.171 mg/g FW), with significant differences between them (Figure 2).

Regarding the flavonoid class, luteolin 7-*O*-glucoside was the most abundant flavonoid across all genotypes, particularly in *L. stoechas* L. (0.643 ± 0.079 mg/g FW) and its cultivar *L. stoechas* ‘Alba’ L. (0.776 ± 0.042 mg/g FW), indicating that the biosynthesis of these compounds is conserved in the cultivars. In addition, flavones have been consistently reported as chemical markers for these species, with high concentrations ranging from ~6 to ~39 μg/g DW documented in *L. stoechas* L. from various locations in Tunisia [4]. Similarly, significant differences were found between *L. latifolia* Medik. and *Lavandula × intermedia* ‘Alba’ Emeric ex Loisel, with concentrations of 1.371 ± 0.325 mg/g FW and 0.593 ± 0.199 mg/g FW, respectively [8], revealing a possible contribution of the *L. latifolia* Medik. species to the hybrid.

#### Antioxidant Potential of Lavandula Phenolic Extracts

The antioxidant activity of phenolic compounds, such as flavonoids and hydroxycinnamic acids, is well established. Their effectiveness is largely determined by their chemical structure, particularly the presence of hydroxyl groups (–OH), conjugated aromatic rings, and other functional groups such as methoxy groups (–OCH_3_), which enable them to act as efficient electron or hydrogen donors capable of neutralizing free radicals [41]. The antioxidant activity of phenolic extracts has attracted considerable attention for industrial applications. Indeed, compound extracts are often prepared using hydroalcoholic systems that benefit their application to biological matrices that could be sensitive to organic solvents [42].

In this regard, the antioxidant activity of *Lavandula* extracts was assessed using the FRAP (ferric-reducing antioxidant power) and the DPPH (2,2-diphenyl-1-picrylhydrazyl) radical scavenging test. In terms of free radical neutralization measured by the DPPH assay, the lowest concentrations required to achieve 50% radical scavenging (EC_50_) were observed for the extracts of *L. latifolia* Medik. (0.17 ± 0.01 µg/mL), *L. stoechas* ‘Alba’ L. (0.18 ± 0.01 µg/mL), *L. stoechas* L. (0.19 ± 0.02 µg/mL), and *L. angustifolia* ‘Krajova’ Mill. (0.18 ± 0.01 µg/mL), with no statistically significant differences among them (*p* > 0.05; Table 2). Furthermore, a Spearman correlation analysis revealed a moderate and statistically significant negative correlation (r = –0.615, *p* < 0.05) between antioxidant activity measured by the DPPH assay and total flavonoid content (Figure S1). Thus, as flavonoid content increases, a lower concentration of extract is required to effectively neutralize free radicals. This result supports the observed trend, whereby *L. stoechas* ‘Alba’ L., *L. stoechas* L., *L. latifolia* Medik., and *L. angustifolia* Mill. exhibited both the highest antioxidant activity and the highest concentrations of flavonoid compounds.

These findings are consistent with several studies reporting DPPH values for aqueous extracts of *L. stoechas* L. (1.78 mg/mL) [29] and *L. angustifolia* Mill. (10.62 ± 0.02 µg/mL) [30], as well as significant Pearson correlations between total flavonoid content and antioxidant activity [13].

Similar results were observed in the FRAP assay for the extracts from the species *L. stoechas* (8.90 ± 0.03 mg/mL), *L. stoechas* ‘Alba’ L. (8.60 ± 0.02 mg/mL), *L. latifolia* Medik. (8.80 ± 0.02 mg/mL), and *L. angustifolia* ‘Krajova’ Mill. (8.70 ± 0.01 mg/mL), which exhibited the highest antioxidant activity in terms of electron-donating capacity, suggesting the potential of the extracts to inhibit oxidative processes involving electron transfer mechanisms [43]. Indeed, a moderate and significant correlation was observed between FRAP and total flavonoid content (r = 0.64, *p* < 0.05). Moreover, several individual compounds exhibited significant correlations with antioxidant activity. Rosmarinic acid showed moderate correlations with both the FRAP assay (r = 0.61, *p* < 0.05) and the DPPH assay (r = –0.67, *p* < 0.05). Likewise, certain 7-*O*-glucosylated flavonoids, such as luteolin 7-*O*-glucuronide and apigenin 7-*O*-glucuronide, displayed moderate and significant correlations with the FRAP assay (r = 0.68 and r = 0.69, respectively). Overall, both FRAP and DPPH assays confirmed the antioxidant activity of the extracts from *L. latifolia* Medik., *L. stoechas* ‘Alba’L., *L. stoechas* L., and *L. angustifolia* ‘Krajova’ Mill.

The variability observed in the polyphenol profile reveals a complementary vision of specialized metabolism that, together with the terpene analysis, enables a more integrated interpretation of the metabolic differences among *Lavandula* species and cultivars.

### 3.2. Terpenes

The biosynthesis of terpenes begins in the cytosol with acetyl coenzyme A via the mevalonate (MVA) pathway, or in plastids via the 2C-methyl-D-erythritol 4-phosphate (MEP) pathway. Both pathways lead to the main terpene precursors: isopentenyl diphosphate (IPP) and its isomer, dimethylallyl diphosphate (DMAPP) [20,44]. The biosynthetic process involves a series of head-to-tail and non-head-to-tail condensations, followed by enzymatic reactions that ultimately give rise to specific terpenes, such as monoterpenes and sesquiterpenes. Furthermore, a subsequent enzyme-mediated step catalyzes the conversion of basic monoterpenes into oxygenated monoterpenes, adding groups such as alcohols, ketones, aldehydes, oxides, or esters [44,45]. In *Lavandula*, monoterpenes represent the most abundant subclass of terpenes, a characteristic associated with the aromatic and antioxidant properties observed across all species [45]. Based on this, it is now possible to establish a chemotaxonomic classification among the known *Lavandula* species and hybrids, which in some cases may serve as a discriminating factor for the selection of a specific species, depending on the intended use or even the desired quality of the essential oil and/or extract to be obtained [46].

**Figure 3:**
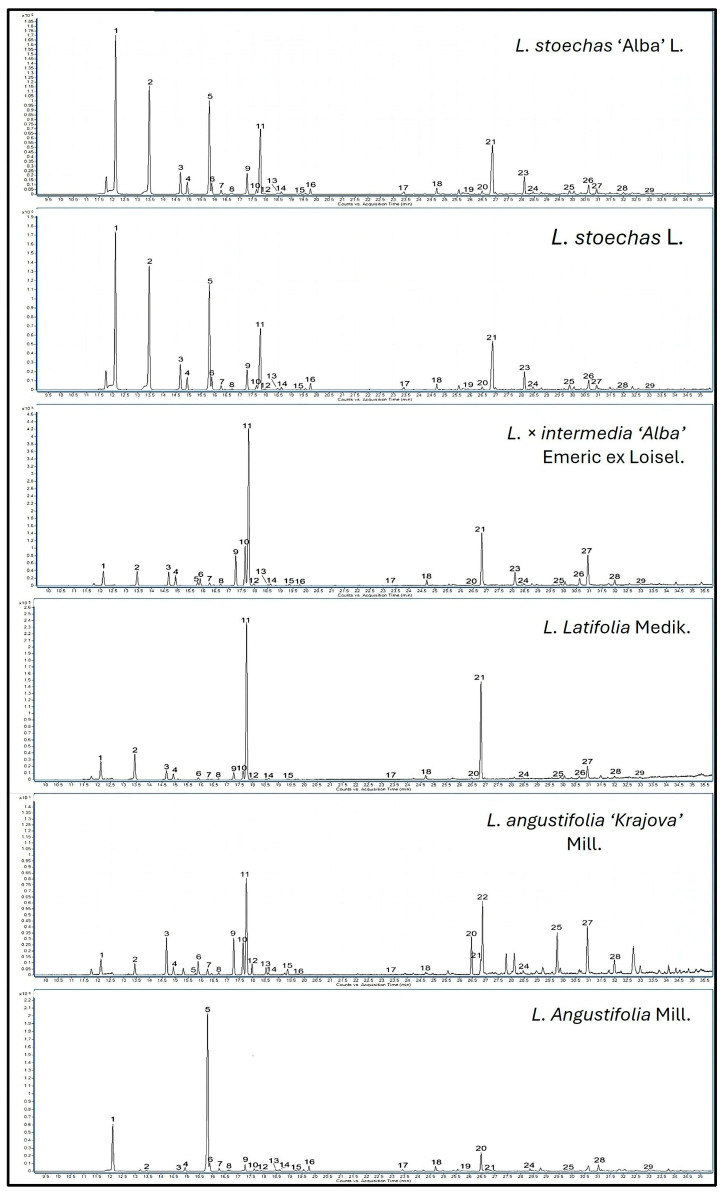
GC chromatogram of the species *L. stoechas* L., *L. latifolia* Medik., *L. angustifolia* Mill., and *L. × intermedia* ‘Alba’ Emeric ex Loisel, and the cultivars *L. stoechas* ‘Alba’ L. and *L. angustifolia* ‘Krajova’ Mill., showing the volatile terpenes identified and quantified. The number of peaks corresponds to the following sequence: 1—*α*-pinene; 2—camphene; 3—*β*-pinene; 4—sabinene; 5—*Δ^3^*-carene; 6—myrcene; 7—*α*-phellandrene; 8—*α*-terpinene; 9—Limonene; 10—*β*-phellandrene; 11—1,8-cineole; 12—*trans-β*-ocimene; 13—*Cis-β*-ocimene; 14—*γ*-terpinene; 15—*p*-cymene; 16—terpinolene; 17—fenchone; 18—*Cis-β*-terpineol; 19—*α*-copaene; 20—linalool; 21—camphor; 22—linalylacetate; 23—lavandulylacetate; 24—4-ol-terpinen; 25—lavandulol; 26—*α*-terpineol; 27—(-)borneol; 28—carvone; 29—myrtenol.

In our study, a total of 29 terpene compounds were identified in the extracts of species, cultivars, and hybrids (Table 2 and Table S2, Figure 3). The results showed that oxygenated monoterpenes constituted the most abundant subclass in the samples, with concentrations ranging from 138.47 to 1922 ng/g FW (approximately 70%), while total monoterpene hydrocarbons ranged from 33.99 to 945.89 ng/g FW (approximately 30%).

**Figure 4:**
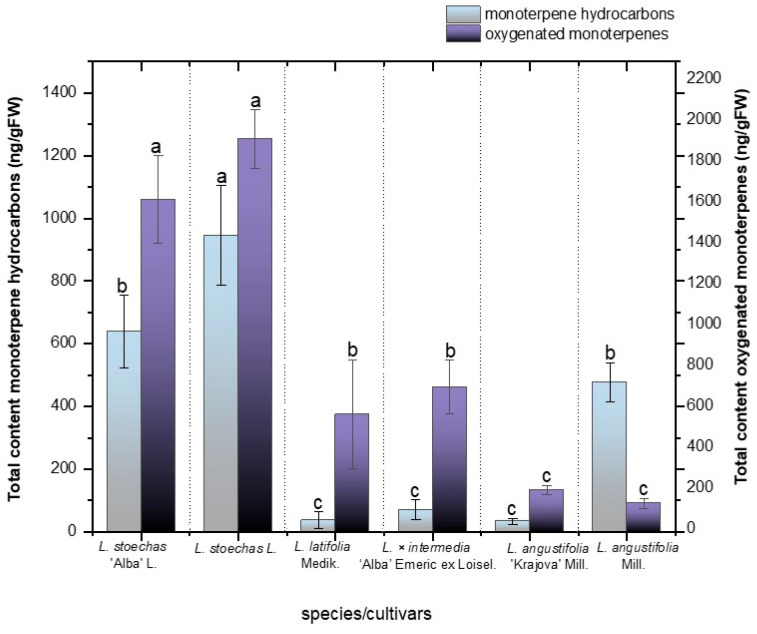
Total content of hydrocarbon monoterpenes and oxygenated monoterpenes in ng/gFW in the species *L. stoechas* L., *L. latifolia* Medik., *L. angustifolia* Mill., and *L. × intermedia* ‘Alba’ Emeric ex Loisel, and the cultivars *L. stoechas* ‘Alba’ L. and *L. angustifolia* ‘Krajova’ Mill. Means with the same letter are not significantly different according to Duncan’s test (*p* > 0.05).

In our study, the highest concentrations of oxygenated monoterpenes were obtained in *L. stoechas* L. (1922.09 ± 144.12 ng/g FW) and its cultivar *L. stoechas* ‘Alba’ L. (1625.96 ± 222.07 ng/g FW, Figure 4), and there were no significant differences between them (Figure 3, *p* < 0.05). These results are in line with several studies confirming the tendency of *L. stoechas* L. to synthesize oxygenated monoterpenes, which have been reported to account for up to 96.7% of its essential oil composition [46]. Correspondingly, *L. × intermedia* ‘Alba’ Emeric ex Loisel and *L. latifolia* Medik. showed statistically similar concentrations of oxygenated monoterpenes, 708.05 ± 130.73 ng/g FW and 574.20 ± 264.49 ng/g FW, respectively, (Figure 4). Furthermore, no significant differences were observed for this class of terpenes between *L. angustifolia* Mill. (203.59 ± 21.86 ng/g FW) and its cultivar *L. angustifolia* ‘Krajova’ Mill. (138.47 ± 25.51 ng/g FW). Notably, certain oxygenated monoterpenes were found to be more abundant in specific species, suggesting that they may serve as markers of distinct chemotypes. This is the case for 1,8-cineole and camphor, the two most abundant compounds among all the species and cultivars studied (Table S2). The biosynthesis of 1,8-cineole is catalyzed by cineole synthase (CINS), which uses geranyl diphosphate (GPP) as a substrate. In contrast, camphor is produced through a multi-step pathway involving bornyl diphosphate synthase (BPPS) and borneol dehydrogenase (BDH). Consistently, *L. stoechas* L. and its cultivar *L. stoechas* ‘Alba’ L. exhibited the highest concentrations of 1,8-cineole (545.26 ± 18.73 and 534.26 ± 45.39 ng/g FW, respectively), followed by the hybrid *L. × intermedia* ‘Alba’ Emeric ex Loisel (311.78 ± 59.37 ng/g FW) (see Table S2). Similarly, for camphor, *L. stoechas* L. (946.89 ± 56.40 ng/g FW), its cultivar *L. stoechas* ‘Alba’ L. (747.93 ± 132.84 ng/g FW), and *L. latifolia* Medik. (307.26 ± 145.22 ng/g FW) exhibited the highest contents among all samples evaluated. These results show a tendency consistent with previously reported values, where camphor was found to be a major compound in *L. latifolia* essential oil, representing 37.3%, while it ranges from 3% to 32% in *L. stoechas* [2,47].

Interestingly, linalyl acetate was identified and quantified only in the cultivar *L. angustifolia* ‘Krajova’ Mill. (7.76 ± 0.63 ng/g FW), highlighting it as a chemotype in this species, which will be discussed in the following sections. In addition, the terpene lavandulol was quantified in higher amounts in *L. stoechas* L. (48.62 ± 12.00 ng/g FW), *L. stoechas* ‘Alba’ L. (25.78 ± 0.92 ng/g FW), and *L. × intermedia* ‘Alba’ Emeric ex Loisel (2.17 ± 0.57 ng/g FW) compared to the other samples. In these species, this monoterpene has been previously reported in their essential oils at proportions of 0.2–3.7% in *L. stoechas*, and in the subspecies *L. stoechas* subsp. *luisieri* (0.3–11.7%) [2].

Likewise, in *L. × intermedia* Emeric ex Loisel, a specialized enzyme, lavandulyl diphosphate synthase (LiLPPS), was identified as a key regulator of lavandulyl diphosphate (LPP) biosynthesis, thereby influencing the production of both lavandulol and lavandulyl acetate. Thus, the tendency of *L. stoechas* and *L. × intermedia* to synthesize lavandulol and subsequently convert it into lavandulyl acetate could explain the presence of this monoterpene only in *L. stoechas* ‘Alba’ (4.22 ± 1.02 ng/g FW), *L. stoechas* L. (6.59 ± 1.52 ng/g FW), and *L. × intermedia* ‘Alba’ (0.97 ± 0.11 ng/g FW), as shown in Table S2.

Regarding monoterpene hydrocarbons, significant differences were found among species and cultivars (Figure 4 and Table S2). *L. stoechas* L. presented the highest monoterpene hydrocarbon content (945 ± 159.26 ng/g FW), followed by its cultivars *L. stoechas* ‘Alba’ L. (633.28 ± 114.33 ng/g FW) and *L. angustifolia* Mill. (477.95 ± 61.83 ng/g FW). In contrast, no significant differences were observed among *L. latifolia* (36.31 ± 26.97 ng/g FW), *L. angustifolia* ‘Krajova’ Mill. (33.93 ± 9.36 ng/g FW), and *L. × intermedia* ‘Alba’ Emeric ex Loisel (70.51 ± 31.32 ng/g FW).

In our study, the abundance of specific compounds within the monoterpene hydrocarbon subclass appears to be species-dependent. Notably, *L. stoechas* and *L. stoechas* ‘Alba’ exhibited the highest concentrations of α-pinene and camphene among all evaluated samples. In *L. stoechas*, α-pinene and camphene reached concentrations of 262.27 ± 38.70 ng/g FW and 311.46 ± 43.73 ng/g FW, respectively. Moreover, in *L. stoechas* ‘Alba’, concentrations were 185.97 ± 35.30 ng/g FW for α-pinene and 192.72 ± 49.19 ng/g FW for camphene (Table S2). Δ^3^-Carene is a monoterpene characteristic of *Lavandula angustifolia*, which in our results is the predominant monoterpene in this species, with a concentration of 302.77 ± 36.43 ng/g FW. These observations suggest a potential species-specific predisposition toward the biosynthesis of this monoterpene.

### 3.3. Metabolite-Based Differentiation of Lavandula Species and Cultivars via PCA

**Figure 5:**
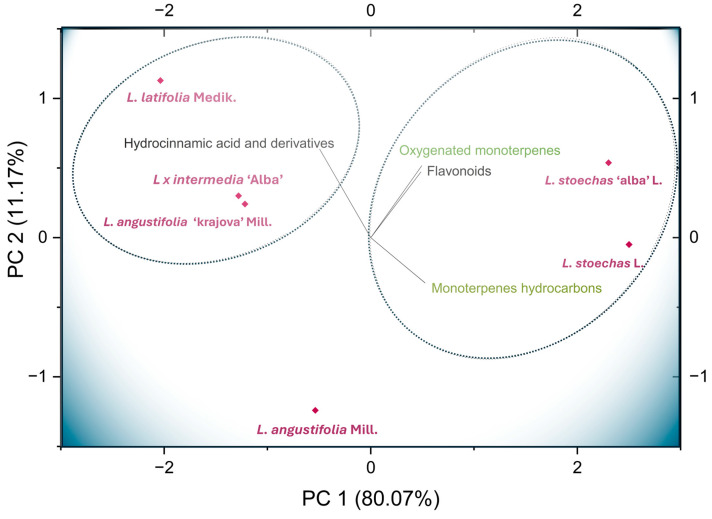
Principal component analysis (PCA) biplot of *Lavandula* species in relation to the composition of flavonoids, hydroxycinnamic acids and their derivatives, monoterpene hydrocarbons, and oxygenated monoterpenes. Red diamonds represent the species *L. stoechas* L., *L. angustifolia* Mill., *L. latifolia* Medik., and *L. × intermedia* ‘Alba’ Emeric ex Loisel, and the cultivars *L. stoechas* ‘Alba’ L. and *L. angustifolia* ‘Krajova’ Mill.

A principal component analysis (PCA) was conducted to simplify the composition data of hydroxycinnamic acids, flavonoids, monoterpene hydrocarbons, and oxygenated monoterpenes across the analyzed species and cultivars, and to identify the metabolites driving species differentiation and clustering patterns. This approach aimed to determine whether species or cultivars could be distinguished based on their secondary metabolite profiles, thereby highlighting their potential applications in specific areas. Figure 5 shows the PCA, which explained 91.24% of the total variance. The first component (PC1, x-axis) accounted for 80.07%, while the second component (PC2, y-axis) explained 11.17%. The compounds with the highest loadings in PC1 were monoterpene hydrocarbons (0.52) and flavonoids (0.48). In contrast, hydroxycinnamic acids and their derivatives (0.63) contributed most strongly to PC2. Oxygenated monoterpenes contributed almost equally to both PC1 (0.49) and PC2 (0.52). These results indicate that the contribution of the compound classes studied accounts for the variability among lavender species. Clear differences and similarities were observed between the species and cultivars according to their metabolite content, allowing the distinction of two main clusters. This division suggests that each species may exhibit optimal performance in specific application areas.

The species *L. stoechas* L. and its cultivar are similar to one another and are distinguished by their high content of oxygenated monoterpenes, monoterpene hydrocarbons, and flavonoids. Thus, they form a cluster 1 (see Figure 5 and Figure S2) that differs from the other *Lavandula* species studied. The second cluster (Figure S2) reflects the similarity between species *L. latifolia* Medik., *L. × intermedia* Emeric ex Loisel, and *L. angustifolia* ‘Krajova’ Mill. in terms of their high content of hydroxycinnamic acid derivatives.

## 4. Discussion

Interspecific variation within the *Lavandula* genus has been extensively documented. These variations could be influenced by various factors, including species, geographical origin, plant parts analyzed, and phenological stages [16]. In this regard, Lopes et al. reported significant differences in the polyphenol content of *L. pedunculata* (Mill.) from different regions of Portugal [40]. Similarly, Lahmar et al. observed significant differences in the flavonoid content of extracts obtained from *L. officinalis* cultivated at different phenological stages, with higher production during the flowering stage [41]. The interspecific variation, also evident among *Lavandula* hybrids in our study, may exclusively derive from genotypic differences since plants were cultivated in the same environment and leaves were collected at the same phenological stage. In the case of the hybrid *L. × intermedia* ‘Alba’ Emeric ex Loisel, the results suggest a possible contribution of the parental species *L. latifolia* to the biosynthesis of HCs, consistent with previous observations comparing hybrids with one of their parental species [30]. The non-significant differences obtained for *L. stoechas* L. and its cultivar ‘Alba’ L. in terms of HC content indicate that the biosynthesis of this class of compounds is conserved in the cultivars of these species [48]. In contrast, the significant differences observed in the HC content between *L. angustifolia* Mill. and its cultivar *L. angustifolia* ‘Krajova’ Mill. are consistent with the findings reported by Dobros et al. for the *L. angustifolia* cultivars Betty’s Blue, Elizabeth, and Hidcote [17].

Furthermore, the antioxidant activity of the polyphenol extracts, evaluated through the DPPH and FRAP assays, indicated that the in vivo effectiveness of hydroxycinnamic acids and flavonoids as antioxidants is not only related to the neutralization of free radicals. A second mechanism of action involves promoting the production of antioxidant enzymes, such as superoxide dismutase and glutathione dismutase, responsible for the degradation of superoxide anions and hydroperoxides, respectively. Specifically, this mechanism allows for the reduction of oxidative processes by strengthening the antioxidant defense system. Upon exposure to oxidative stress conditions, living organisms (humans, animals, and plants) experience a dysregulation between the production of reactive oxygen species (ROS) and their neutralization mechanisms. Polyphenols stimulate antioxidant enzymes, including superoxide dismutase and glutathione peroxidase, enhancing their expression and activity. Through these enzymatic pathways, ROS are efficiently neutralized before they can initiate chain oxidation reactions. Consequently, the structural integrity of lipids, proteins, and nucleic acids is preserved, maintaining cellular homeostasis and preventing oxidative damage [13,49,50]. The results obtained from antioxidant assays (DPPH and FRAP) confirm consistent trends across the analyzed *Lavandula* species and cultivars. Extracts with higher contents of flavonoids and hydroxycinnamic acid derivatives exhibited greater antioxidant activity in both assays, whereas those with lower levels of these compounds showed reduced activity. Minor differences in absolute values observed in each assay could be attributed to the distinct sensitivities of the methods to different molecular mechanisms (hydrogen atom donation in the DPPH assay versus electron transfer in the FRAP assay).

In relation to terpene concentrations in the species, cultivar, and hybrid of *Lavandula* analyzed, the predominance of oxygenated compounds has been previously reported in certain Lavandula species and attributed to the activity of specific enzymes responsible for the production of these particular classes of compounds [20,44]. Furthermore, evidence has shown that the abundance of these compounds is directly associated with the plant’s resistance to biotic stressors, such as phytophagous insects and pathogenic microorganisms [5,46]. Additionally, the results on terpenes showed that some species and cultivars possess specialized enzymes related to the biosynthesis of chemotypes. In this regard, the elevated production of 1,8-cineole has been specifically associated with *L. stoechas* L. and *L. × intermedia* ‘Alba’ Emeric ex Loisel and is catalyzed by cineole synthase (CINS), using the geranyl diphosphate (GPP) as a substrate [2,51], while camphor accumulation has been linked to *L. stoechas* L. and *L. latifolia* Medik. involving bornyl diphosphate synthase (BPPS) and borneol dehydrogenase (BDH) [42].

Moreover, the identification of linalyl acetate only in the cultivar *L. angustifolia* ‘Krajova’ Mill. agrees with the results previously reported by Dušková et al. [52], who found high concentrations ranging from 7.20% to 30.40% in the essential oil of this cultivar. In addition, linalyl acetate has been reported at levels ranging from 9.08% to 24.45% in the essential oils of seven new Ukrainian cultivars of *L. angustifolia* Mill. [53]. Indeed, this oxygenated monoterpene is synthesized only by some *L. angustifolia* cultivars and varieties, such as cv. ‘Krajova’ and var. Hemus, through the acetylation of linalool catalyzed by alcohol acyltransferase (AAT), using acetyl-CoA as the acetyl donor [53,54]. Thus, determining the linalool/linalyl acetate ratio is crucial for specific applications of essential oils and extracts from *Lavandula* species, as it significantly influences the aromatic and therapeutic quality of both the extract and the essential oil [54]. Meanwhile, the identification of lavandulol in *L. stoechas* L. is of particular importance because it functions as the direct precursor of lavandulyl acetate. The biosynthetic pathway involves alcohol acyltransferase (AAT) in the presence of acetyl-CoA; this enzyme catalyzes the conversion of lavandulol into lavandulyl acetate. The efficiency of this reaction varies across *Lavandula* species and cultivars, which can explain the differences in lavandulyl acetate content observed in our study [2,54].

The highest concentrations of monoterpene hydrocarbons found in *L. stoechas*, compared to *L. angustifolia* Mill. and *L. × intermedia* ‘Alba’ Emeric ex Loisel, have also been observed in essential oils of these species, with a higher proportion of monoterpenes and yields of approximately 6.4% and 5.6% for *L. stoechas* L. and *L. angustifolia* Mill., respectively [55]. Among the monoterpene hydrocarbons, it has also been possible to identify certain chemotypes, such as carene, which is associated with *Lavandula angustifolia* Mill., representing approximately 0.2% of its total essential oil [3].

In light of the results obtained, the analysis of terpene profiles across *Lavandula* samples revealed considerable variability, with *L. stoechas* L. showing notable distinction. Moreover, certain terpenes were identified as potential markers for distinguishing specific chemotypes of *Lavandula* species, highlighting differences in the biosynthesis of oxygenated compounds.

The results obtained from the principal component analysis showed a clear grouping into two clusters, the first composed of the species *L. stoechas* L. and its cultivar, which are distinguished by higher chemical variability and greater metabolite abundance, suggesting their potential as optimal candidates for applications requiring high antioxidant activity due to their high content of flavonoids. However, this species may not be ideal for cosmetic or perfumery applications due to its high content of oxygenated monoterpenes. This class of compounds is known to reduce essential oil quality in cosmetics because they are chemically unstable and can degrade into by-products with strong or undesirable odors [20,55]. In contrast, the oil exhibits excellent antibacterial activity [20]. The second cluster is characterized by its hydroxycinnamic acid composition, making these species excellent candidates for applications requiring antioxidant, antibacterial, and anti-inflammatory activity [7,20].

## 5. Conclusions

In conclusion, our results revealed clear variability in terms of secondary metabolites composition among the six *Lavandula* genotypes analyzed. The identification of compounds showed that the main phenolic constituents correspond to glycosylated derivatives of coumaric, caffeic, and ferulic acids, as well as luteolin and apigenin derivatives. In terms of quantification, *L. latifolia* Medik. exhibited the highest total polyphenol content, which was associated with the strongest antioxidant activity detected for this species. In addition, *L. stoechas* L. and its cultivar *L. stoechas* ‘Alba’ L. displayed the highest flavonoid concentrations, also correlating with their excellent antioxidant capacity. Regarding terpenes, oxygenated monoterpenes were the most abundant across all species/cultivars. The *L. stoechas* L. species and its cultivar had the highest concentrations of oxygenated monoterpenes and total monoterpenes. In addition, some chemotypes, such as linalyl acetate in *L. angustifolia* Mill. and lavandulol in *L. stoechas* L., were identified and quantified.

The variability observed across compound classes highlights the potential of these species as valuable sources for specific applications across various industrial sectors. Moreover, the chemical characterization enabled the identification of specific marker compounds in certain species based on their phenolic profiles, as well as distinct chemotypes within the terpene class. Overall, the results suggest that the genetic background within the *Lavandula* genus plays a key role in regulating the biosynthesis of secondary metabolites, generating intra- and interspecific variations that may support their selection for targeted applications across specific sectors.

## Figures and Tables

**Table 2:** Results of antioxidant activity measured using FRAP and DPPH assays, from phenolic extracts of *Lavandula* species (*L. stoechas* L., *L. latifolia* Medik., and *L. angustifolia* Mill.), cultivars (*L. stoechas* ‘Alba’ L. and *L. angustifolia* ‘Krajova’ Mill.), and the hybrid *L.* × *intermedia* ‘Alba’ Emeric ex Loisel. Means with the same letter are not significantly different according to Duncan’s test (*p* > 0.05).

Species/Cultivars	EC_50_ (in µg/mL)	FRAP (mM Ferrous Equiv.)
*L*. *stoechas* ‘Alba’ L.	0.18 ± 0.01 ^c^	8.60 ± 0.02 ^b^
*L*. *stoechas* L.	0.19 ± 0.02 ^c^	8.90 ± 0.03 ^b^
*L. × intermedia* ‘Alba’ Emeric ex Loisel	0.27 ± 0.02 ^a^	6.80 ± 0.01 ^c^
*L. latifolia* Medik.	0.17 ± 0.01 ^d^	8.80 ± 0.02 ^b^
*L. angustifolia* ‘Krajova’ Mill.	0.18 ± 0.01 ^c^	8.70 ± 0.01 ^b^
*L. angustifolia* Mill.	0.22 ± 0.02 ^b^	7.00 ± 0.01 ^c^
Positive control	0.10 ± 0.012 ^d^	61.17 ± 5.92 ^a^

## Data Availability

The original contributions presented in this study are included in the article/Supplementary Materials. Further inquiries can be directed to the corresponding author.

## Supplementary Materials

The following supporting information can be downloaded at: https://www.mdpi.com/article/10.3390/metabo16010034/s1, Table S1: Hydrocinnamic acid and flavonoid content in mg/g DW of *L. stoechas* L, *L*. *latifolia* Medik., *L. angustifolia* Mill., and *L.* × *intermedia* ‘Alba’ Emeric ex Loisel, and the cultivars *L. stoechas* ‘Alba’ L. and *L. angustifolia* ‘Krajova’ Mill. Table S2: Summary of terpene composition and content (μg/g DW) of *L. stoechas* L., *L. latifolia* Medik., *L. angustifolia* Mill., and *L.* × *intermedia* ‘Alba’ Emeric ex Loisel, and the cultivars *L*. *stoechas* ‘Alba’ L. and *L. angustifolia* ‘Krajova’ Mill. Figure S1: Spearman correlation matrix of hydroxycinnamic acid derivatives, flavonoids, and FRAP and DPPH antioxidant activity quantified in *Lavandula* species and cultivars. Figure S2: Hierarchical cluster analysis (HCA) of six Lavandula species and cultivars based on their phytochemical profiles. The dendrogram was constructed using the Euclidean distance and average linkage. Figures S3–S21: Tentative identifications of peaks (HPLC-DAD); (a) Example chromatogram of a sample with the corresponding UV spectrum acquired by HPLC-DAD. (b) UV spectrum acquired by HPLC-ESI-QTOF. (c) HPLC-ESI-QTOF mass spectrum showing the precursor ion at *m*/*z*.
