# Supplementary material for: From Ornamental Value to Antioxidant Activity: Comparative Phytochemistry of Lavandula Species and Cultivars
Source: Metabolites. 2025 Dec 30;16(1):34. doi: 10.3390/metabo16010034 (PMC12844421; doi:10.3390/metabo16010034)
Supplement: Supplementary file 1 [file metabolites-16-00034-s001.zip › metabolites-4018721-supplementary.pdf]

From ornamental value to antioxidant activity: comparative phytochemistry of *Lavandula* species and cultivars

Andrea Baptista [1,2], Cecilia Brunetti [1,2*], Dalila Pasquini [2], Luana Beatriz Dos Santos Nascimento[2], Cassandra Detti [2], Francesco Ferrini [2,1], Beltrami Sara[2] and Antonella Gori [2,1].

[1] Institute for Sustainable Plant Protection, National Research Council of Italy (CNR), Via Madonna del Piano 10, I-50019 Sesto Fiorentino, Florence, Italy; andreacarolinabaptistaruiz@cnr.it (A.B.); cecilia.brunetti@cnr.it (C.B).

[2] Department of Agriculture, Food, Environment and Forestry, University of Florence, Viale delle Idee 30, I-50019 Sesto Fiorentino, Florence, Italy; dalila.pasquini@unifi.it (D.P.); luanabeatriz.dossantosnascimento@unifi.it (LB.SN); cassandra.detti@unifi.it (C.D.) francesco.ferrini@unifi.it (F.F.); sara.beltrami@unifi.it; antonella.gori@unifi.it (A.G.).

* Corresponding author: cecilia.brunetti@cnr.it; Tel.: +39-055-457-4024; I-50019 Sesto Fiorentino, Florence, Italy.

Table S1. Hydrocinnamic acid derivatives and flavonoids content in mg/g FW of *L. stoechas* L., *L. latifolia* Medik., *L. angustifolia* Mill., and *L.* × *intermedia* 'Alba' Emeric ex Loisel., and the cultivars: *L. stoechas* 'Alba' L., and *L. angustifolia* 'Krajová' Mill. Values are reported as mean ± standard deviation of three replicates. Means with the same letter are not significantly different by Duncan test ($p > 0.05$).

					**Concentration (mg/g FW)**					
**n°**	Rt (min)	Identification tentative	$[M-H]^-$ m/z	UV max (nm)	*L. Stoechas* 'Alba' L.	*L. stoechas* L.	*L. latifolia* Medik.	*L.* × *intermedia* 'Alba'	*L. angustifolia* 'Krajová' Mill.	*L. angustifolia* Mill.
					**Hydroxycinnamic acid derivatives**					
**1**	22.56	1-o-Caffeoylglucose	341	300, 326	0.007±0.001^{b}	0.009±0.003^{b}	0.007±0.004^{b}	0.063±0.034^{b}	0.282±0.152^{a}	0.016±0.001^{b}
**2**	26.10	trans-p-Coumaric acid 4-*O*-glucoside	325	285	0.030±0.002^{c}	0.024±0.007^{c}	2.252±0.469^{a}	1.883±0.243^{a}	1.159±0.213^{b}	0.098±0.009^{c}
**3**	28.92	Feruloyltartaric acid[a]	325	328	0.191±0.020^{a}	0.212±0.053^{a}	nd	nd	nd	nd
**4**	29.27	Ferulic acid *O*-glucoside[b]	355	280, 305	nd	nd	0.205±0.043^{b}	0.672±0.239^{a}	0.842±0.076^{a}	0.380±0.028^{b}
**5**	30.06	Caffeic acid acetylhexoside[a]	387	328	0.038±0.010^{a}	0.038±0.002^{a}	0.009±0.003^{b}	nd	nd	nd
**7**	32.05	*o*-Coumaric acid 2-*O*-glucoside	325	275, sh313	0.061±0.009^{c}	0.054±0.007^{c}	2.174±0.262^{a}	0.199±0.084^{b}	0.190±0.014^{b}	0.175±0.055^{b}
**8**	35.63	Ferulic acid-*O*-glucoside	355	318-320	0.046±0.001^{c}	0.036±0.004^{c}	0.260±0.096^{b}	0.423±0.096^{b}	0.536±0.045^{a}	0.315±0.024^{b}
**14**	43.93	Rosmarinic acid	359	328	0.157±0.016^{a}	0.138±0.017^{a}	0.146±0.192^{a}	nd	0.113±0.092^{a}	nd
**16**	46.56	Salvianolic acid B[a]	717	sh 334	0.067±0.014^{a}	0.051±0.006^{b}	nd	nd	nd	nd
**18**	50.32	Unknow 2[a]	501	326	0.223±0.005^{a}	0.197±0.016^{b}	nd	nd	nd	nd
**19**	56.94	Unknow 3	727	322	0.012±0.003^{c}	0.017 ±0.003^{c}	0.252 ± 0.196^{b}	0.618 ± 0.257^{a}	0.737 ± 0.404^{a}	0.730±0.163^{a}
		**Total**			**0.833±0.082^{d}**	**0.777 ±0.118^{d}**	**5.306±1.265^{a}**	**3.858±0.954^{b}**	**3.858±0.280^{b}**	**1.713±0.280^{c}**
					**Flavonoids**					
**6**	31.11	Apigenin C-hexoside	431	334	0.066±0.017^{c}	0.042±0.015^{c}	0.020±0.006^{c}	0.179±0.023^{b}	0.357±0.109^{a}	0.211±0.018^{bc}
**9**	37.22	Luteolin 7-*O*-glucuronide	461	256, 267sh, 347	0.256±0.022^{a}	0.184±0.013^{b}	0.128 ±0.068	nd	0.040±0.024^{c}	0.004±0.002^{c}
**10**	37.72	Luteolin 7-*O*-glucoside	447	256, 267sh, 350	0.776±0.042^{a}	0.643±0.079^{a}	0.252±0.198^{b}	0.136±0.002^{b}	0.659±0.365^{a}	0.036±0.015^{b}

**11**	41.23	Apigenin-7-*O*-Glucoside	431	267, 332	0.207±0.021 [a]	0.137±0.007[b]	0.029±0.011[c]	0.020±0.012[c]	0.012±0.004[c]	0.209±0.047 [a]
**12**	41.85	Apigenin 7- *O*-glucuronide	445	268, 333	0.645±0.039[a]	0.507±0.021[b]	0.070±0.019[c]	0.015±0.001[d]	0.043±0.020[c]	0.017±0.007[d]
**13**	42.48	Methylluteolin-*O*-glucuronide	475	350	0.351±0.030[a]	0.285±0.023[b]	0.002±0.001[c]	nd	nd	nd
**15**	45.82	Apigenin 7-(6''-acetylglucoside) [a]	473	334	0.236±0.021[a]	0.158±0.013[b]	0.037±0.003[c]	0.016±0.004[d]	0.016±0.009[d]	0.023±0.004[cd]
**17**	49.45	Unknow 1[b]	331	298,312	nd	nd	0.833 ± 0.019[a]	0.227 ± 0.157[b]	0.161 ± 0.089[b]	0.203±0.010[b]
		**Total flavonoids**			**2.537±0.192**[a]	**1.956±0.171**[b]	**1.371± 0.325**[c]	**0.593±0.199**[d]	**1.288±0.621**[c]	**0.703±0.104**[d]
		**Total Polyphenol content**			**3.372±0.274**[c]	**2.734±0.290**[c]	**6.686±1.593**[a]	**4.457±1.157**[b]	**5.173±1.636**[b]	**2.424±0.385**[c]

[a] Identified and quantified only in *L. stoechas* and *L. stoechas* 'Alba'; [b] identified and quantified only in *L. latifolia,* L. × *intermedia* 'Alba', *L. angustifolia* and *L. angustifolia 'Krajová'*.

Table S2. Terpene composition and content (ng/g FW) of the species *L. stoechas* L., *L. latifolia* Medik., *L. angustifolia* Mill., and *L. × intermedia* 'Alba' Emeric ex Loisel., and the cultivars: *L. stoechas* 'Alba' L., and *L. angustifolia* 'Krajová' Mill. Values are reported as mean ± standard deviation of three replicates. Means with the same letter are not significantly different by Duncan test (p > 0.05).

			Concentration (ng/g FW)					
**n°**	Rt (min)	Putative identification	*L. Stoechas* 'Alba' L.	*L. stoechas* L.	*L. latifolia* Medik.	*L. × intermedia* 'Alba'	*L. angustifolia* 'Krajová' Mill.	*L. angustifolia* Mill.
			**monoterpenes hydrocarbons**					
**1**	12.13	α-pinene	185.97±35.30^{b}	262.27± 38.70^{a}	8.50± 7.22^{d}	7.65±3.63^{d}	1.82±0.18^{d}	56.60±8.77^{c}
**2**	13.44	camphene	192.72 ±49.19^{b}	311.46 ±43.73^{a}	11.44±5.75^{c}	7.51±1.30^{c}	1.34±0.25^{c}	2.09±0.55
**3**	14.67	β-pinene	22.94±2.06^{b}	35.34±8.76^{a}	3.76 ±3.33^{c}	5.67±2.81^{c}	3.48±0.35^{c}	0.28±0.055^{c}
**4**	14.93	sabinene	12.69±1.08^{a}	14.30±0.32^{a}	2.04±1.65^{c}	4.31±2.63^{b}	0.89±0.29^{c}	4.23±0.73^{b}
**5**	15.78	Δ^3-carene	114.19±17.93^{c}	175.63±38.03^{b}	nd	2.08±1.43^{d}	0.80±0.90^{d}	302.77±36.43^{a}
**6**	15.89	myrcene	12.06±1.24^{b}	17.64±3.20^{a}	0.60±0.41^{d}	3.80±2.62^{c}	2.31±1.54^{c}	11.18±0.49^{b}
**7**	16.26	α-phellandrene	4.27±0.59^{b}	5.20±0.09^{a}	0.16±0.12^{d}	1.01±0.45^{c}	0.74±0.33^{c}	4.93±0.28^{a}
**8**	16.68	α-terpinene	3.20±0.34^{a}	3.23±0.17^{a}	0.51±0.38^{d}	1.20±0.42^{c}	0.51±0.01^{d}	2.76±0.04^{b}
**9**	17.26	Limonene	44.44±0.56^{a}	52.52±12.28^{a}	6.81±6.86^{c}	23.18±10.38^{bc}	6.41±0.58^{c}	15.45±1.46^{b}
**10**	17.62	β-phellandrene	1.47±0.08^{b}	1.82±0.57^{b}	0.47±0.28^{b}	5.05±3.34^{a}	2.41±2.41^{b}	0.89±0.08^{c}
**12**	17.97	Trans -β-ocimene	0.67±0.23^{b}	0.72±0.07^{b}	0.19±0.12^{d}	0.47±0.08^{c}	1.41±0.01^{a}	0.26±0.08^{d}
**13**	18.52	Cis- β-ocimene	5.81±1.02^{c}	8.58±0.67^{b}	nd	0.68±0.19^{e}	2.69±0.83^{d}	12.04±1.39^{a}
**14**	18.62	γ-terpinene	2.07±0.35^{b}	2.79±0.75^{a}	0.33±0.23^{c}	0.51±0.32^{c}	0.16±0.01^{c}	1.89±0.18^{b}
**15**	19.35	p-cymene	13.88±2.01^{b}	13.82±1.55^{b}	1.50±0.56^{d}	6.46±1.12^{c}	8.73±1.32^{c}	29.42±6.78^{a}
**16**	19.78	terpinolene	22.63±3.79^{c}	40.50±10.45^{a}	nd	0.93±0.39^{d}	0.23±0.30^{d}	33.16±4.55^{b}
		**Total**	**639.10±114.33^{b}**	**945.89±159.26^{a}**	**36.31±26.97^{c}**	**70.51±31.32^{c}**	**33.93±9.36^{c}**	**477.95± 61.83^{b}**
			**oxygenated monoterpenes**					
**11**	17.76	1,8-cineole	534.26±45.39^{a}	545.26±18.73^{a}	196.44±88.95^{c}	311.78±59.37^{b}	38.055±6.52^{d}	nd
**17**	23.48	fenchone	138.46±32.94^{b}	180.15±19.84^{a}	10.16±1.64^{c}	11.37±0.37^{c}	11.35±0.75^{c}	130.18±2.70^{b}
**18**	24.69	Cis-β-terpineol	15.53±1.14^{b}	19.06±4.14^{a}	3.25±2.74^{d}	5.17±2.75^{d}	0.39±0.10^{d}	11.55±1.42^{c}
**19**	25.94	α-copaene	0.04±0.03^{d}	0.31±0.28^{a}	nd	nd	nd	0.18±0.06^{b}
**20**	26.46	linalool	6.44±0.57^{b}	7.69±2.47^{b}	0.47±0.10^{c}	0.45±0.01^{c}	7.65±1.46^{b}	39.05±9.02^{a}
**21**	26.83	camphor	747.93±132.84^{b}	946.89±56.40^{a}	307.26±145.22^{c}	198.13±47.66^{d}	2.50±0.36^{e}	3.68±4.17^{e}

**22**	26.90	linalylacetate	nd	nd	nd	nd	7.76±0.63 ^{a}	nd
**23**	28.10	lavandulylacetate	4.22±1.02 ^{b}	6.59±1.52 ^{a}	nd	0.97±0.11 ^{c}	nd	nd
**24**	28.44	4-ol-terpinen	7.69±0.83 ^{b}	9.28±0.63 ^{a}	0.70±0.44 ^{d}	1.50±0.19 ^{d}	0.10±0.01 ^{e}	3.30±0.96 ^{c}
**25**	29.87	lavandulol	25.78±0.92 ^{b}	48.62±12.00 ^{a}	0.74±0.21 ^{c}	2.17±0.57 ^{c}	1.10±0.69 ^{c}	0.31±0.30 ^{d}
**26**	30.63	α-terpineol	24.25±2.45 ^{b}	42.19±12.27 ^{a}	1.27±0.52 ^{c}	6.33±2.38 ^{c}	nd	nd
**27**	30.94	(-)borneol	105.84±1.46 ^{b}	105.16±14.15 ^{b}	49.68±23.89 ^{c}	159.11±15.02 ^{a}	62.15±13.85 ^{c}	nd
**28**	31.99	carvone	12.24±1.8327 ^{a}	6.37±1.25 ^{b}	3.98±3.46 ^{c}	10.45±2.2027 ^{a}	7.38±1.11 ^{b}	2.80±0.05 ^{c}
**29**	32.97	myrtenol	3.21±0.60 ^{b}	4.46±0.39 ^{b}	0.19±0.01 ^{c}	0.57±0.05 ^{c}	nd	12.49±3.15 ^{a}
		**Total**	**1625.96±222.07** ^{b}	**1922.09± 144.12** ^{a}	**574.20± 264.49** ^{c}	**708.05± 130.73** ^{c}	**138.47± 25.51** ^{d}	**203.59± 21.86** ^{d}

		(1)	(2)	(3)	(4)	(5)	(6)	(8)	(9)	(15)	(16)	(19)	(20)	THCA der	(7)	(10)	(11)	(12)	(13)	(14)	(17)	(18)	TFC	FRAP	DPPH
Rho de Spearman	(1)-1-o-Caffeoylglucose	1.0																							
	(2)- trans-p-Coumaric acid 4-glucoside	0.4	1.0																						
	(3)-Caffeoyl-feruloyltartaric acid	,529*	,598**	1.0																					
	(4)-Feruloyltartaric acid	-,567*	-,781**	-,749**	1.0																				
	(5)-Ferulic acid O-glucoside	,872**	,652**	,645**	-,811**	1.0																			
	(6)-Caffeic acid acetylhexoside	-,619**	-,468*	-,605**	,807**	-,729**	1.0																		
	(8)-o-Coumaric acid 2-O-glucoside	0.2	,837**	,709**	-,808**	,534*	-,619**	1.0																	
	(9)-Ferulic acid-O-glucoside	,841**	,639**	,713**	-,800**	,923**	-,646**	,575*	1.0																
	(15)-Rosmarinic acid	-,469*	-0.3	-0.2	,545*	-,480*	,736**	-0.4	-0.4	1.0															
	(16)-Salvianolic acid B	-,812**	-,546*	-,513*	,788**	-,862**	,729**	-0.4	-,792**	,514*	1.0														
	(19)-Rosmarinic acid der	-,761**	-0.1	0.0	0.4	-,696**	,531*	0.0	-,579*	,496*	,775**	1.0													
	(20)-unknown 1	,759**	,523*	,659**	-,768**	,805**	-,882**	,556*	,730**	-,655**	-,785**	-,554*	1.0												
	Total Hydrocinnamic acid der	0.4	,915**	,781**	-,781**	,631**	-0.5	,882**	,690**	-0.2	-,478*	0.0	,542*	1.0											
	(7)-Apigenin-O-hexosylpentosyl	,785**	0.1	0.4	-0.4	,737**	-,578*	0.0	,649**	-0.3	-,672**	-,736**	,608**	0.1	1.0										
	(10)-Luteolin 7-glucuronide	-,752**	-,508*	-0.3	,724**	-,793**	,801**	-0.4	-,692**	,826**	,837**	,812**	-,772**	-0.4	-,535*	1.0									
	(11)-Luteolin 7-O-glucoside	-0.2	-0.4	-0.3	,618**	-0.4	,721**	-,523*	-0.3	,881**	,472*	0.3	-,635**	-0.3	-0.1	,715**	1.0								
	(12)-Apigenin-7-O-glucoside	-,585*	-,641**	-0.4	0.5	-,608**	0.2	-0.4	-,639**	0.1	,575*	0.3	-0.4	-,614**	-0.2	0.4	0.0	1.0							
	(13)-Apigenin 7- O-glucuronide	-,657**	-,552*	-0.4	,800**	-,776**	,828**	-,567*	-,713**	,813**	,831**	,705**	-,796**	-0.5	-,486*	,952**	,787**	0.3	1.0						
	(14)-Methylluteolin-O-glucuronide	-,635**	-,657**	-,533*	,827**	-,751**	,893**	-,641**	-,616**	,741**	,725**	,498*	-,849**	-,572*	-0.4	,855**	,727**	0.3	,831**	1.0					
	(17)-Apigenin 7-(6''-acetylglucoside)	-,771**	-,689**	-,689**	,885**	-,825**	,853**	-,670**	-,825**	,697**	,853**	,541*	-,846**	-,659**	-,473*	,857**	,670**	,534*	,863**	,878**	1.0				
	(18)-.Ferulic acid der	0.3	,774**	,736**	-,811**	,473*	-,653**	,902**	,538*	-0.4	-0.5	0.1	,669**	,851**	0.0	-0.4	-,603**	-0.4	-,547*	-,702**	-,725**	1.0			
	Total Flavonoids content	-,637**	-,631**	-0.4	,793**	-,759**	,810**	-,562*	-,670**	,870**	,775**	,624**	-,779**	-,486*	-0.4	,946**	,856**	0.4	,950**	,874**	,872**	-,561*	1.0		
	FRAP	-0.4	-0.2	0.0	,503*	-,492*	,595**	-0.2	-0.4	,609**	0.5	,600**	-,472*	-0.1	-0.5	,677**	,537*	-0.1	,693**	,598**	0.4	-0.2	,641**	1.0	
	DPPH	,470*	0.0	-0.2	-0.1	0.4	-0.4	-0.1	0.2	-,665**	-0.5	-,715**	0.4	-0.2	0.4	-,684**	-,501*	0.0	-,590*	-0.4	-0.3	-0.1	-,615**	-,539*	1.0

Figure S1. Spearman correlation matrix of hydroxycinnamic acid derivatives, flavonoids and FRAP and DPPH antioxidant activity quantified in *Lavandula* species and cultivars. The asterisks indicate statistical significance ($p < 0.05$).

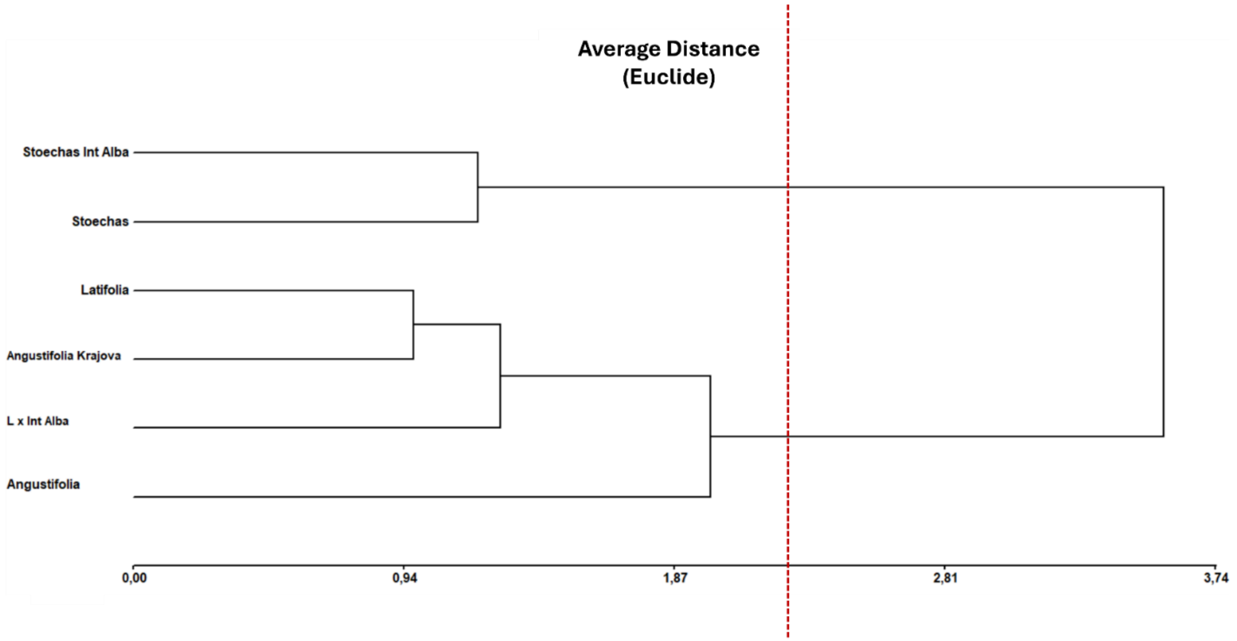

Figure S2. Hierarchical cluster analysis (HCA) of the *Lavandula* species and cultivars based on their phytochemical profiles. The dendrogram was constructed using Euclidean distance and average linkage.

Tentative identification of the peak 1: 1-*o*-Caffeoylglucose (m/z 342; [M-H]$^-$ = 341 m/z, fragment ; [M-H-162]$^-$ = m/z 179.

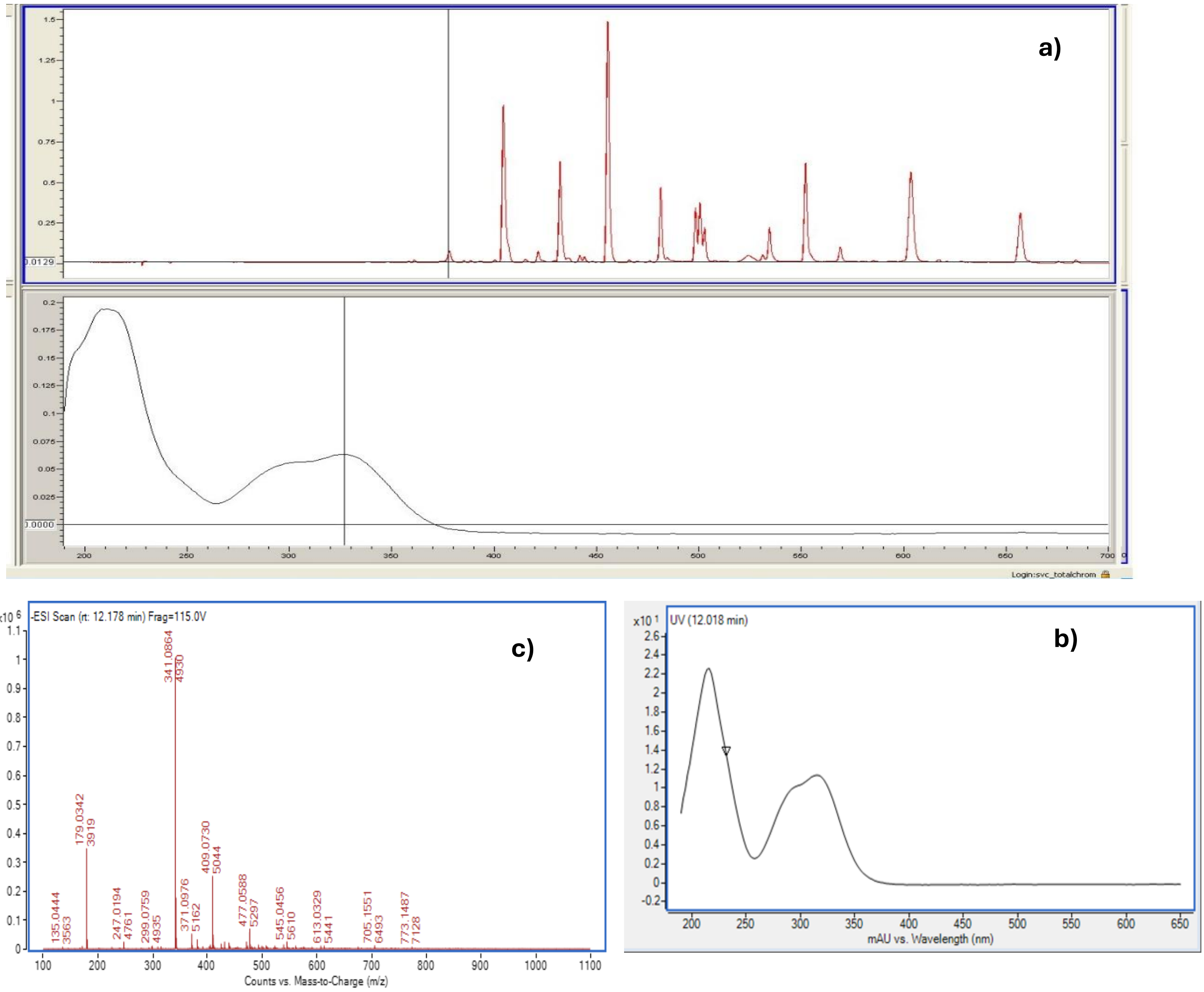

Figure S3. Tentative identification of Peak 1 (HPLC-DAD), Rt = 22.56 min. a) Example chromatogram of a sample with the corresponding UV spectrum acquired by HPLC-DAD. b) UV spectrum of Peak 1 acquired by HPLC-ESI-QTOF. c) HPLC-ESI-QTOF mass spectrum showing the precursor ion at m/z

Tentative identification of the peak 2 : *trans-p*-Coumaric acid 4-glucoside (m/z 326; [M-H]$^-$ = 325 m/z)

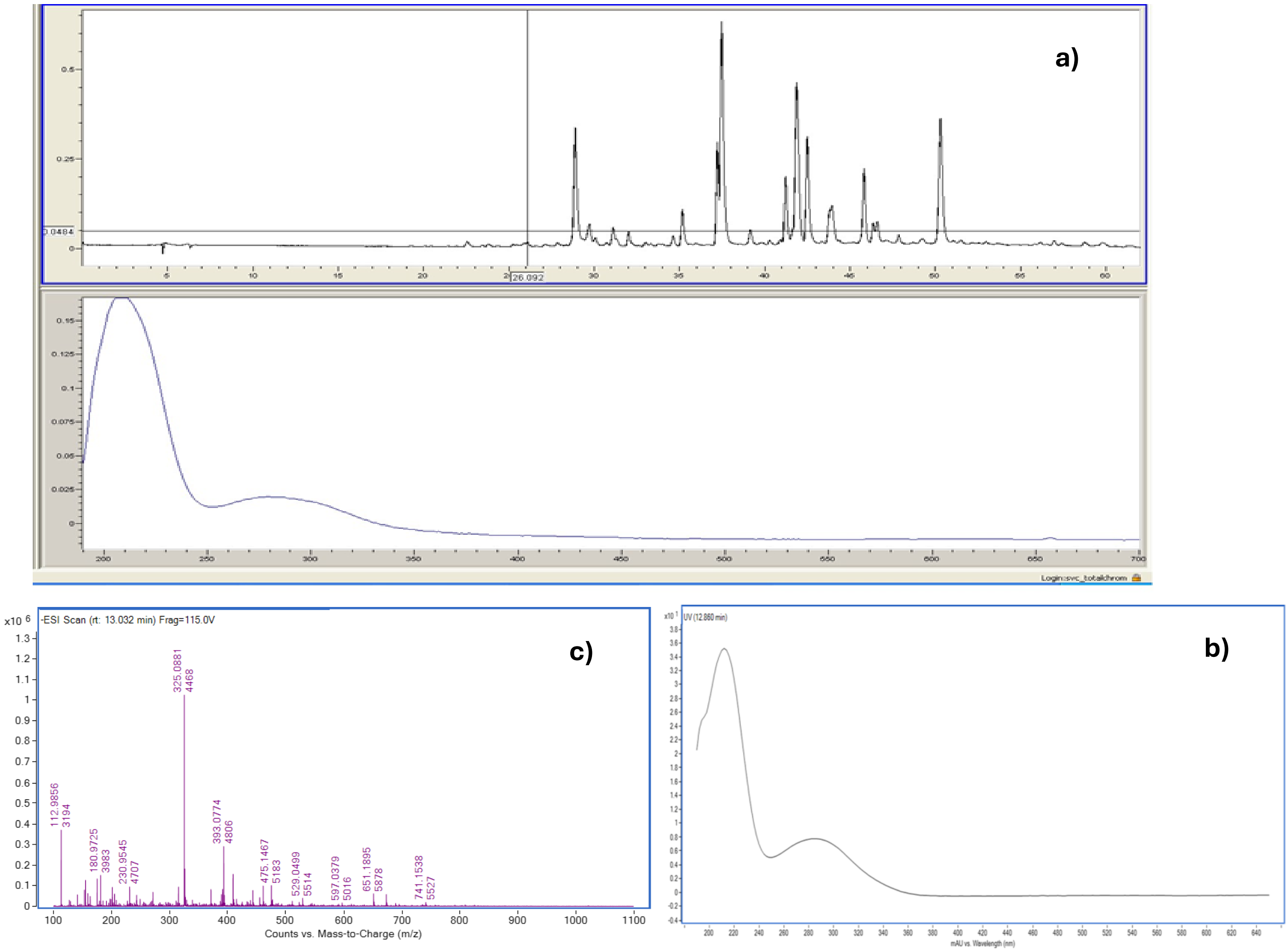

Figure S4. Tentative identification of Peak 2 (HPLC-DAD), Rt = 26.10 min. a) Example chromatogram of a sample with the corresponding UV spectrum acquired by HPLC-DAD. b) UV spectrum of Peak 2 acquired by HPLC-ESI-QTOF. c) HPLC-ESI-QTOF mass spectrum showing the precursor ion at m/z

Tentative identification of the peak 3: m/z [M-H]$^-$ = 325 m/z Feruloyl tartar acid, fragment m/ z 193

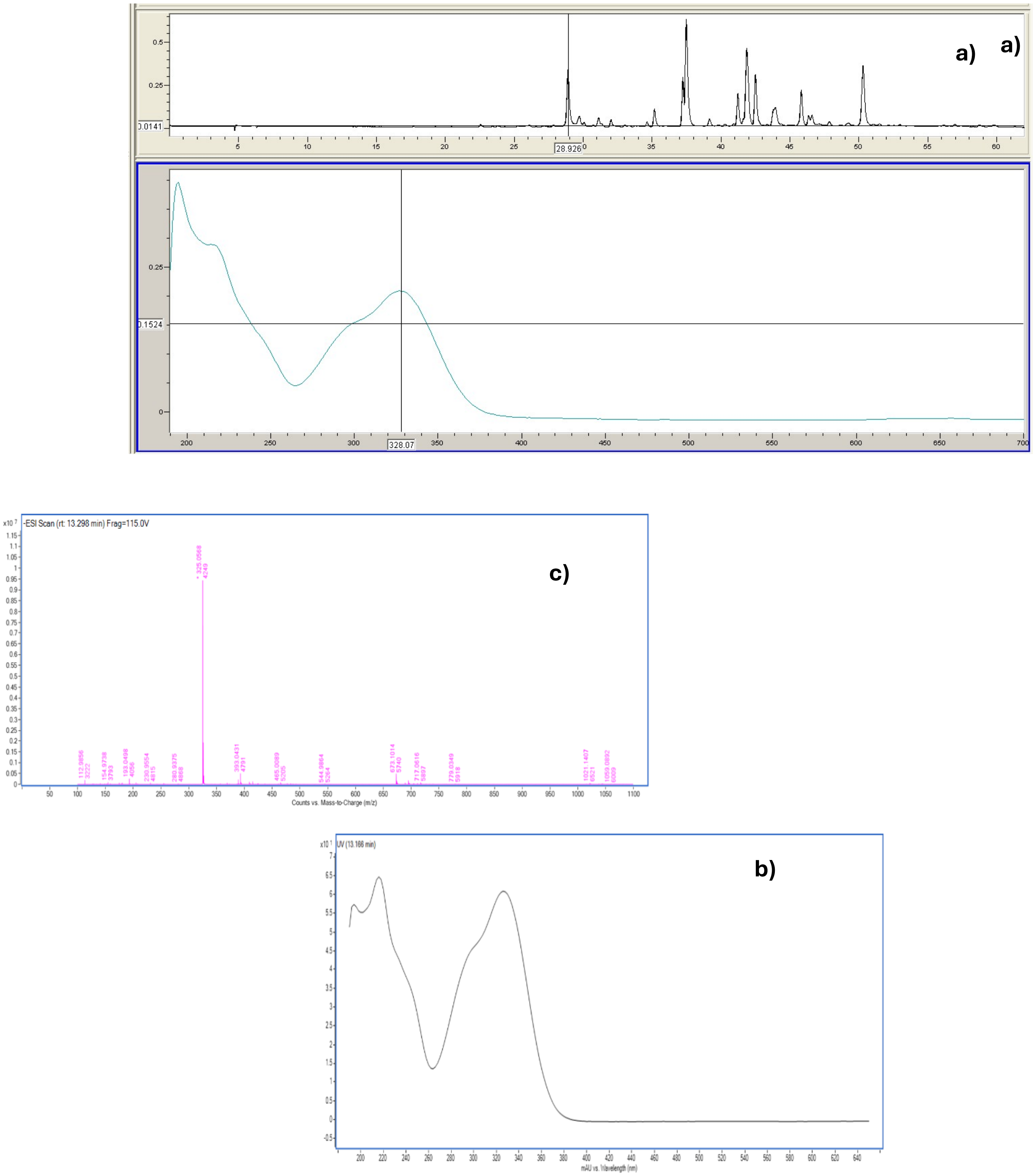

Figure S5. Tentative identification of Peak 3 (HPLC-DAD), Rt = 28.92 min. a) Example chromatogram of a sample with the corresponding UV spectrum acquired by HPLC-DAD. b) UV spectrum of Peak 3 acquired by HPLC-ESI-QTOF. c) HPLC-ESI-QTOF mass spectrum showing the precursor ion at m/z

Tentative identification of the peak 4 (Ferulic acid O-glucoside) : [M-H]⁻ = 355 m/z, fragment m/z 193

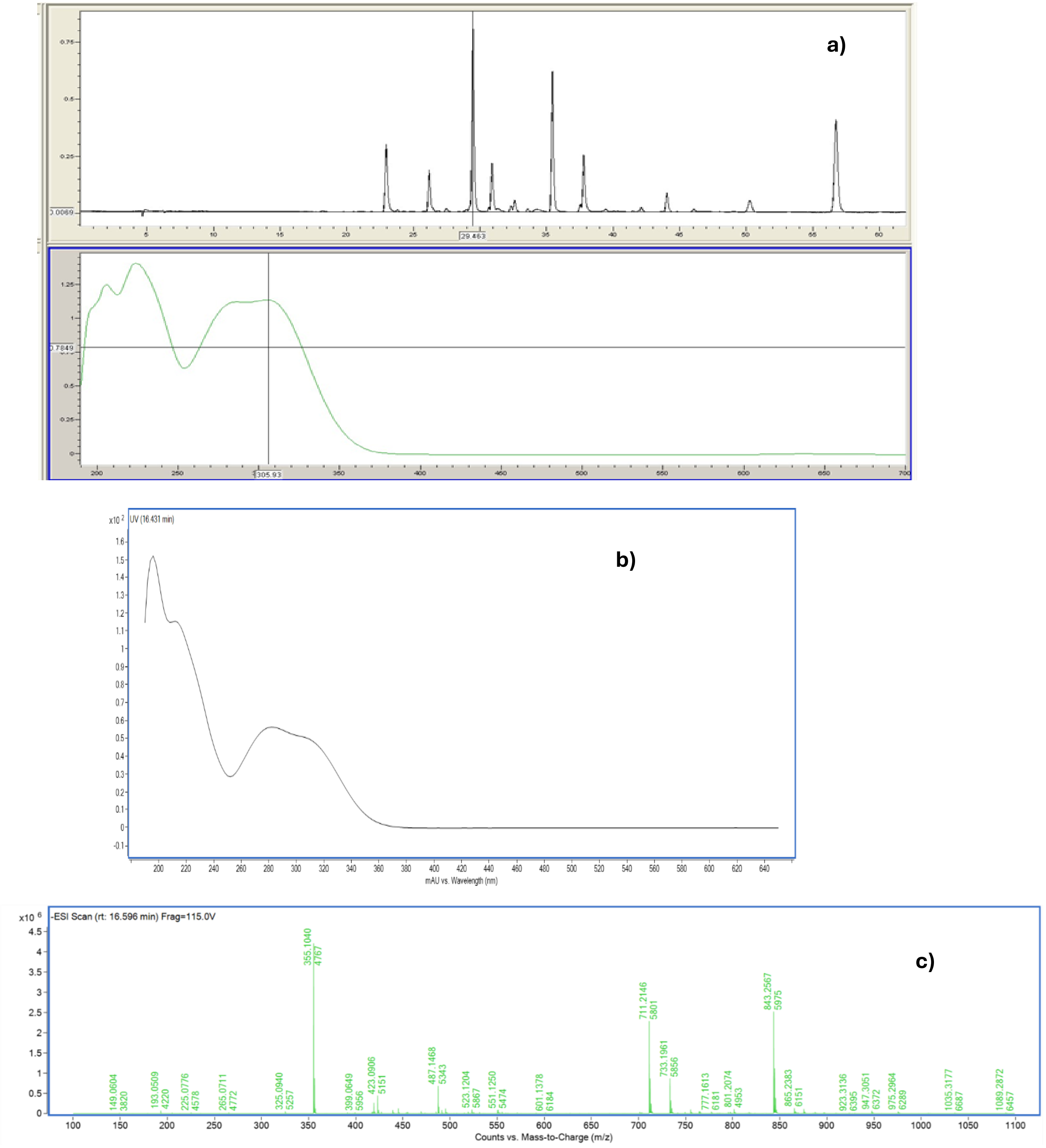

Figure S6. Tentative identification of Peak 4 (HPLC-DAD), Rt = 29.2 min. a) Example chromatogram of a sample with the corresponding UV spectrum acquired by HPLC-DAD. b) UV spectrum of Peak 4 acquired by HPLC-ESI-QTOF. c) HPLC-ESI-QTOF mass spectrum showing the precursor ion at m/z.

Tentative identification of the peak 5 (Caffeic acid acetylhexoside) : [M-H]= 387 m/z

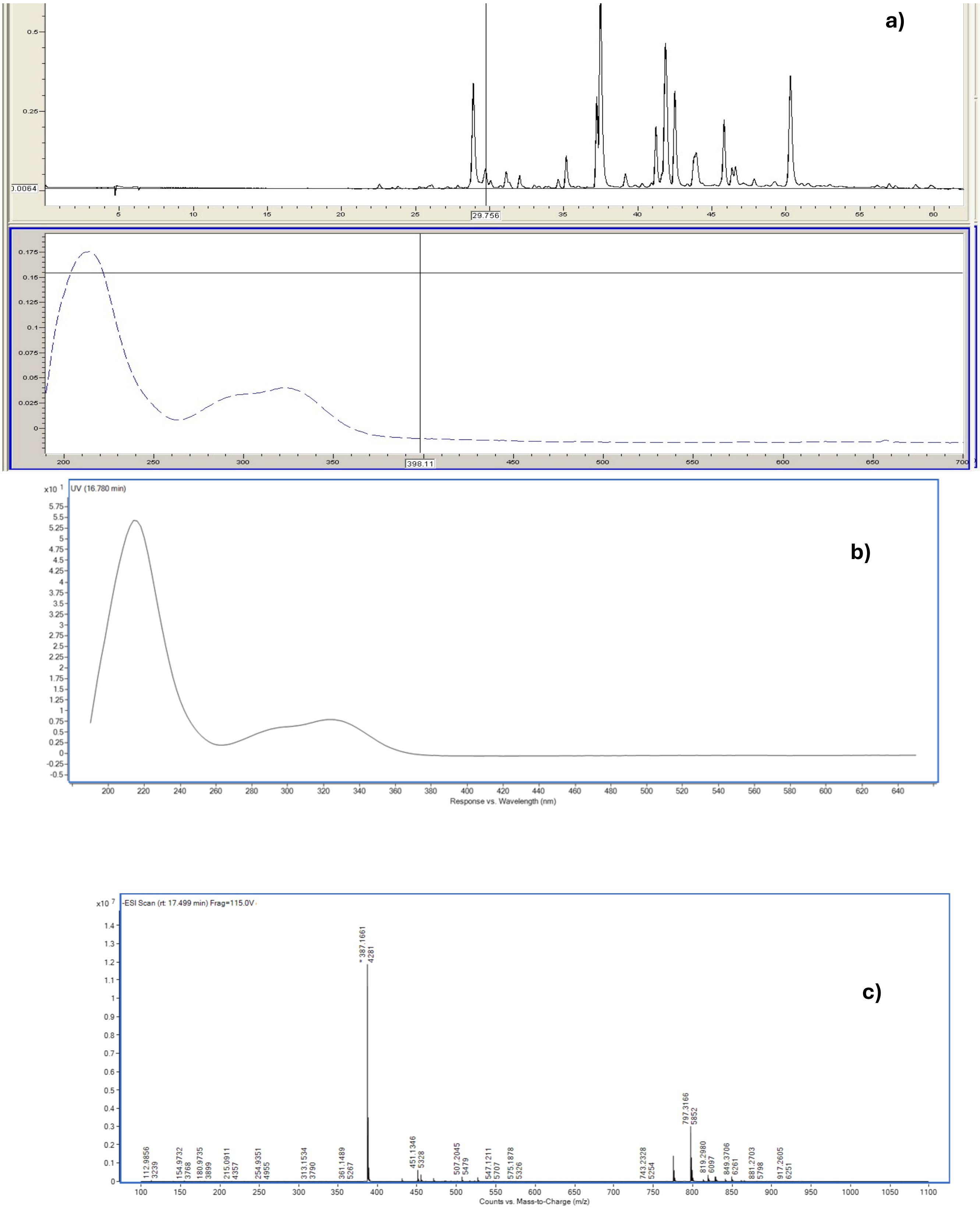

Figure S7. Tentative identification of Peak 5 (HPLC-DAD), Rt = 30.0 min. a) Example chromatogram of a sample with the corresponding UV spectrum acquired by HPLC-DAD. b) UV spectrum of Peak 5 acquired by HPLC-ESI-QTOF. c) HPLC-ESI-QTOF mass spectrum showing the precursor ion at m/z.

Tentative identification of the peak 6 (Apigenin C-hexoside) : [M-H]= 431 m/z

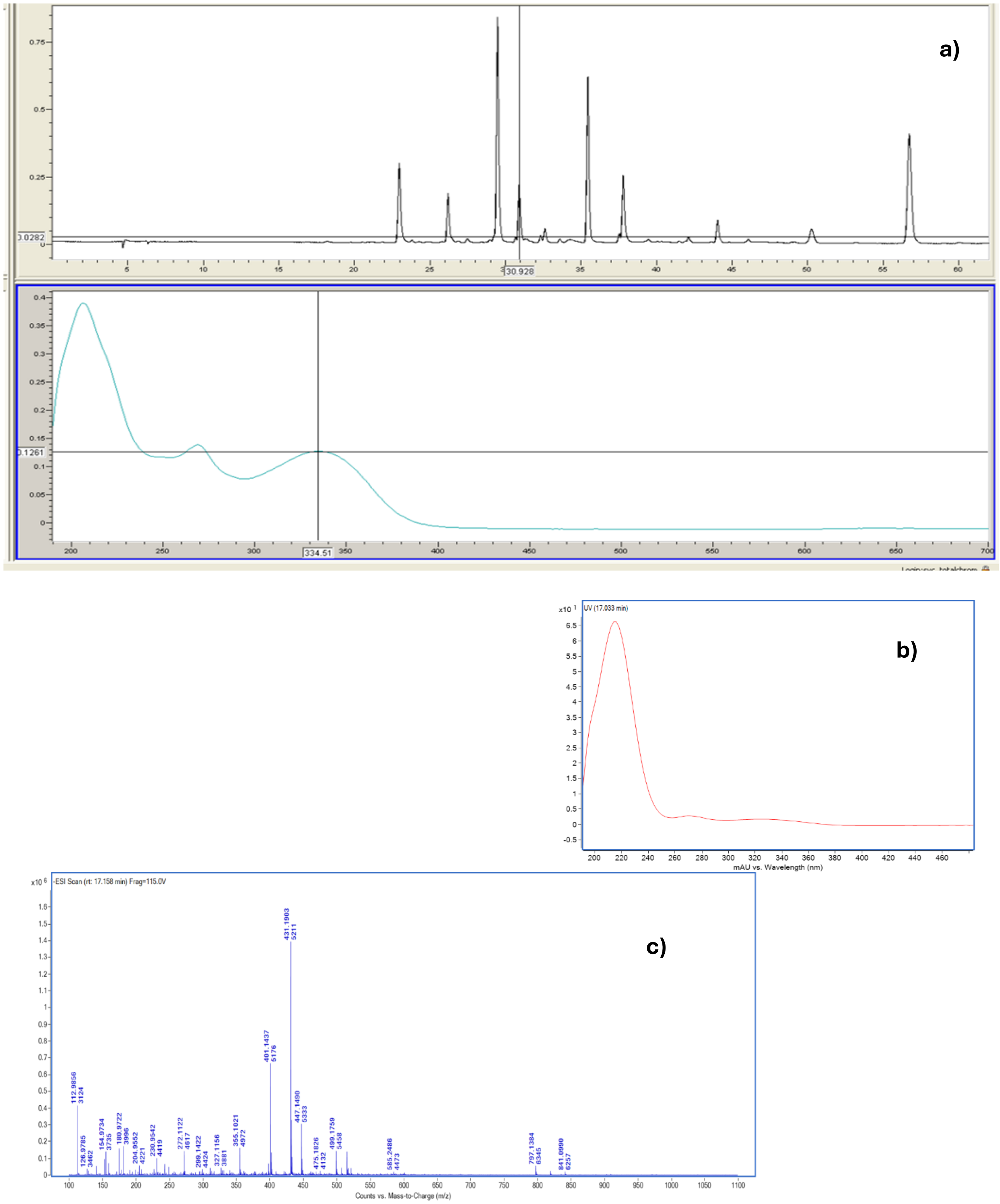

Figure S8. Tentative identification of Peak 6 (HPLC-DAD), Rt = 31.00 min. a) Example chromatogram of a sample with the corresponding UV spectrum acquired by HPLC-DAD. b) UV spectrum of Peak 6 acquired by HPLC-ESI-QTOF. c) HPLC-ESI-QTOF mass spectrum showing the precursor ion at m/z.

Tentative identification of the peak 7 (*o*-Coumaric acid 2-*O*-glucoside) : [M-H]= 325 m/z , fragment [M-H-162]$^-$ = m/z 163 , dimer =m/z 651

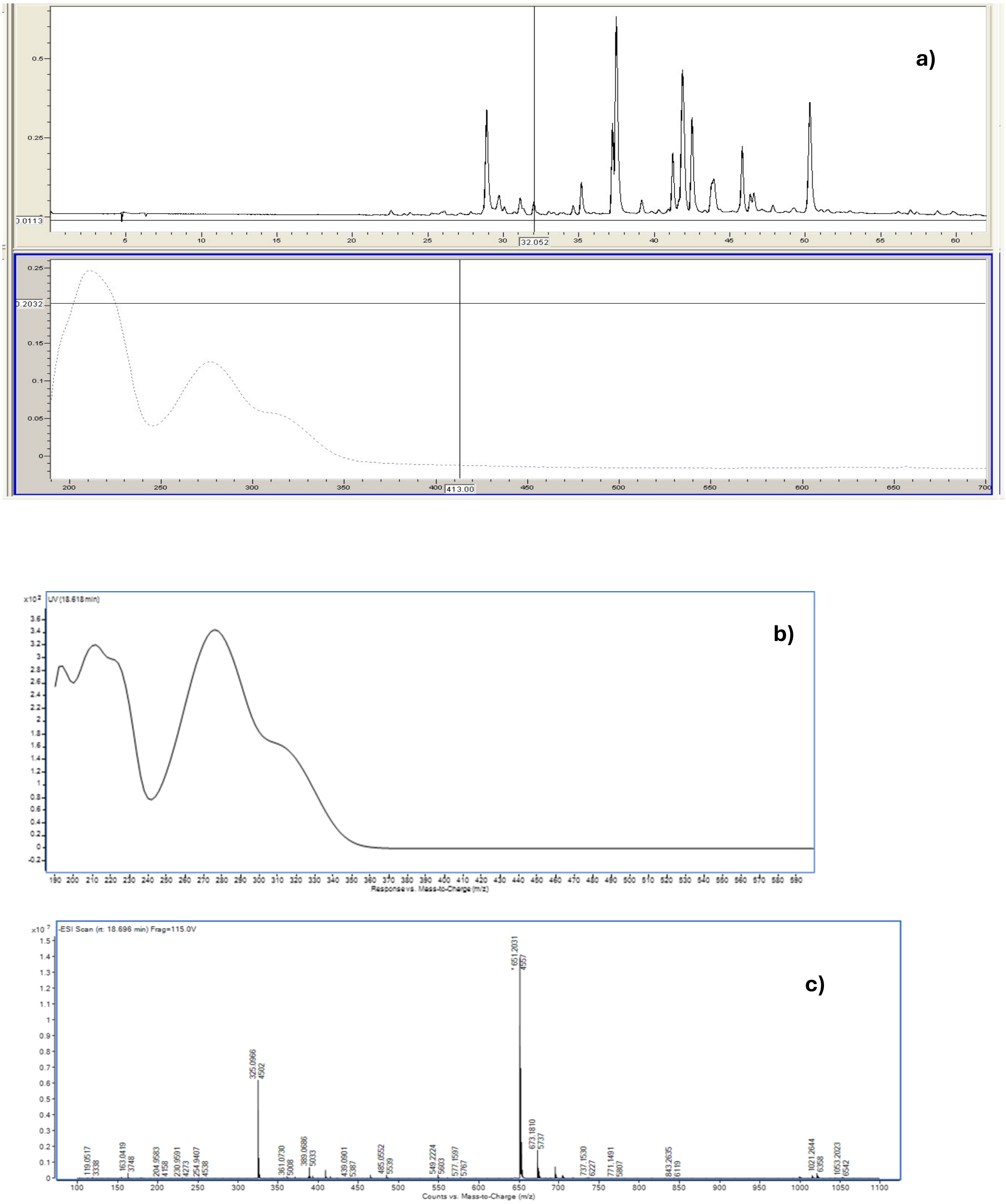

Figure S9. Tentative identification of Peak 7 (HPLC-DAD), Rt = 32.05 min. a) Example chromatogram of a sample with the corresponding UV spectrum acquired by HPLC-DAD. b) UV spectrum of Peak 7 acquired by HPLC-ESI-QTOF. c) HPLC-ESI-QTOF mass spectrum showing the precursor ion at m/z.

Tentative identification of Peak 8 (ferulic acid-O-glucoside): [M–H]⁻ = m/z 355.
Fragments: [M–H–162]⁻ = m/z 193; dimer of 355 = m/z 711.

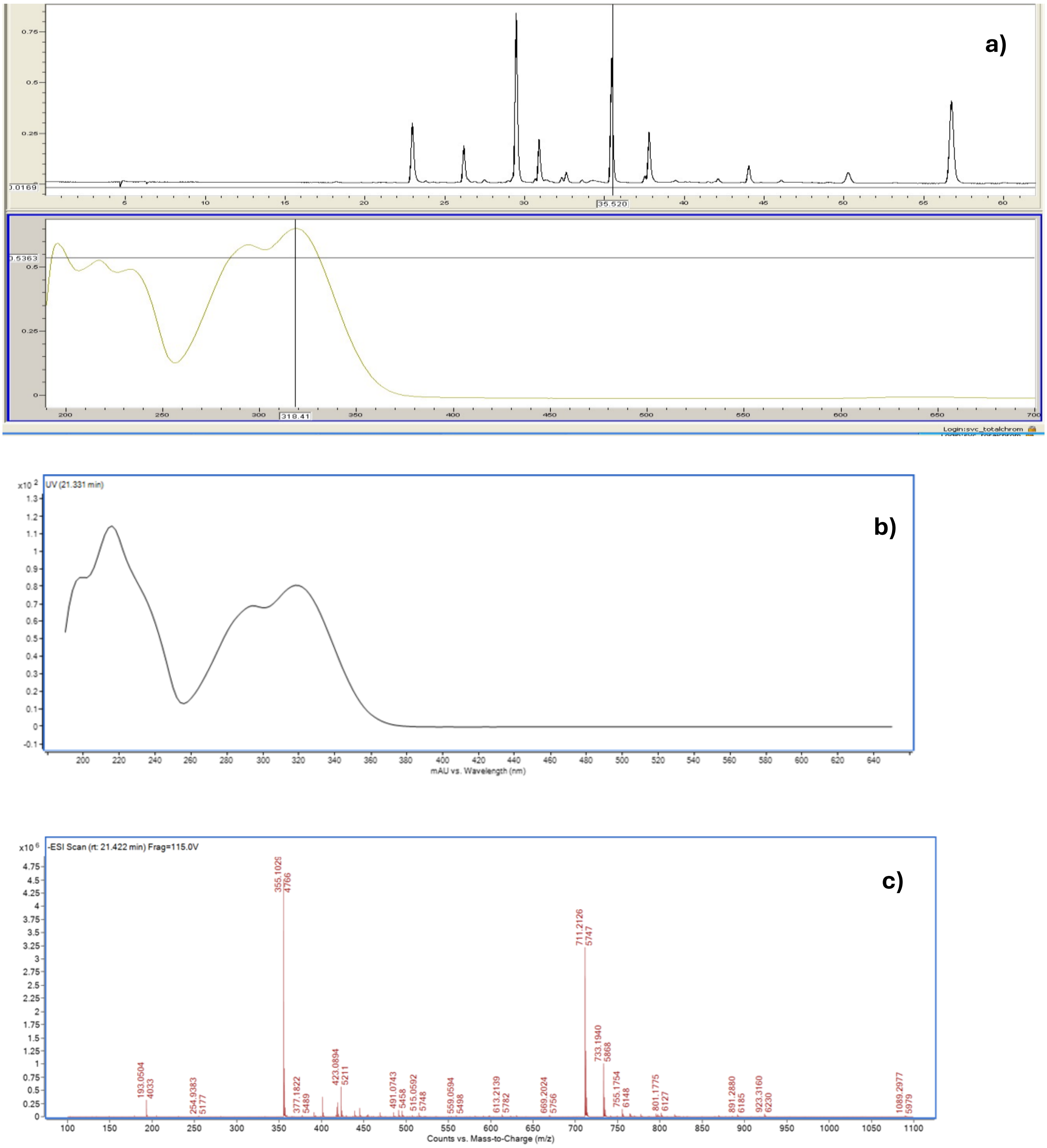

Figure S10. Tentative identification of Peak 8 (HPLC-DAD), Rt = 35.52 min. a) Example chromatogram of a sample with the corresponding UV spectrum acquired by HPLC-DAD. b) UV spectrum of Peak 8 acquired by HPLC-ESI-QTOF. c) HPLC-ESI-QTOF mass spectrum showing the precursor ion at m/z.

Tentative identification of Peak 9 (Luteolin 7-glucuronide) : [M-H]$^-$ = 461 m/z . Fragment : [M-H-162]$^-$ = 285 , dimer : m/z 923

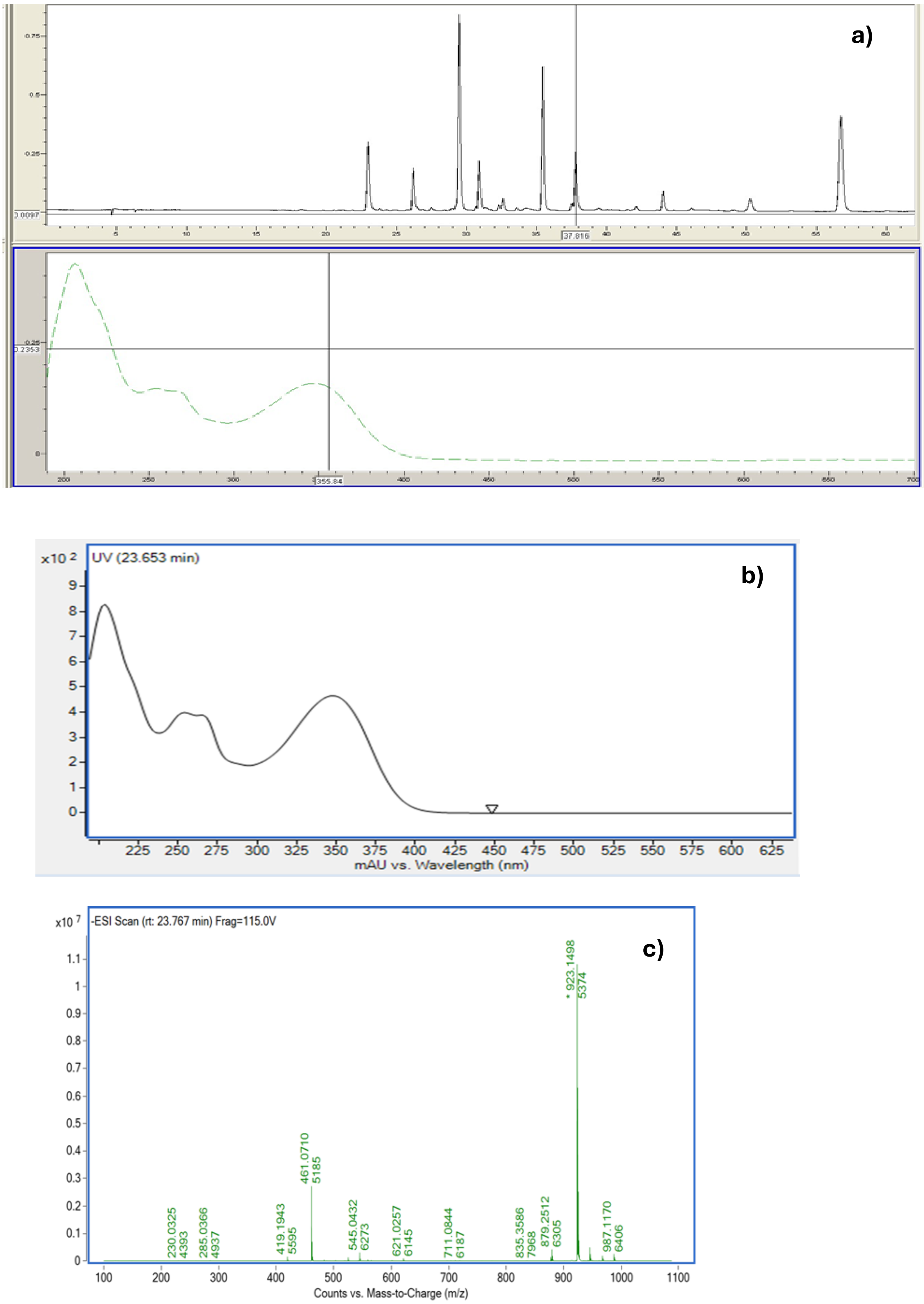

Figure S11. Tentative identification of Peak 9 (HPLC-DAD), Rt = 37.22 min. a) Example chromatogram of a sample with the corresponding UV spectrum acquired by HPLC-DAD. b) UV spectrum of Peak 9 acquired by HPLC-ESI-QTOF. c) HPLC-ESI-QTOF mass spectrum showing the precursor ion at m/z.

Tentative identification of Peak 10 (Luteolin 7-O-glucoside) : [M-H]= 447 m/z, dimer: m/z 895

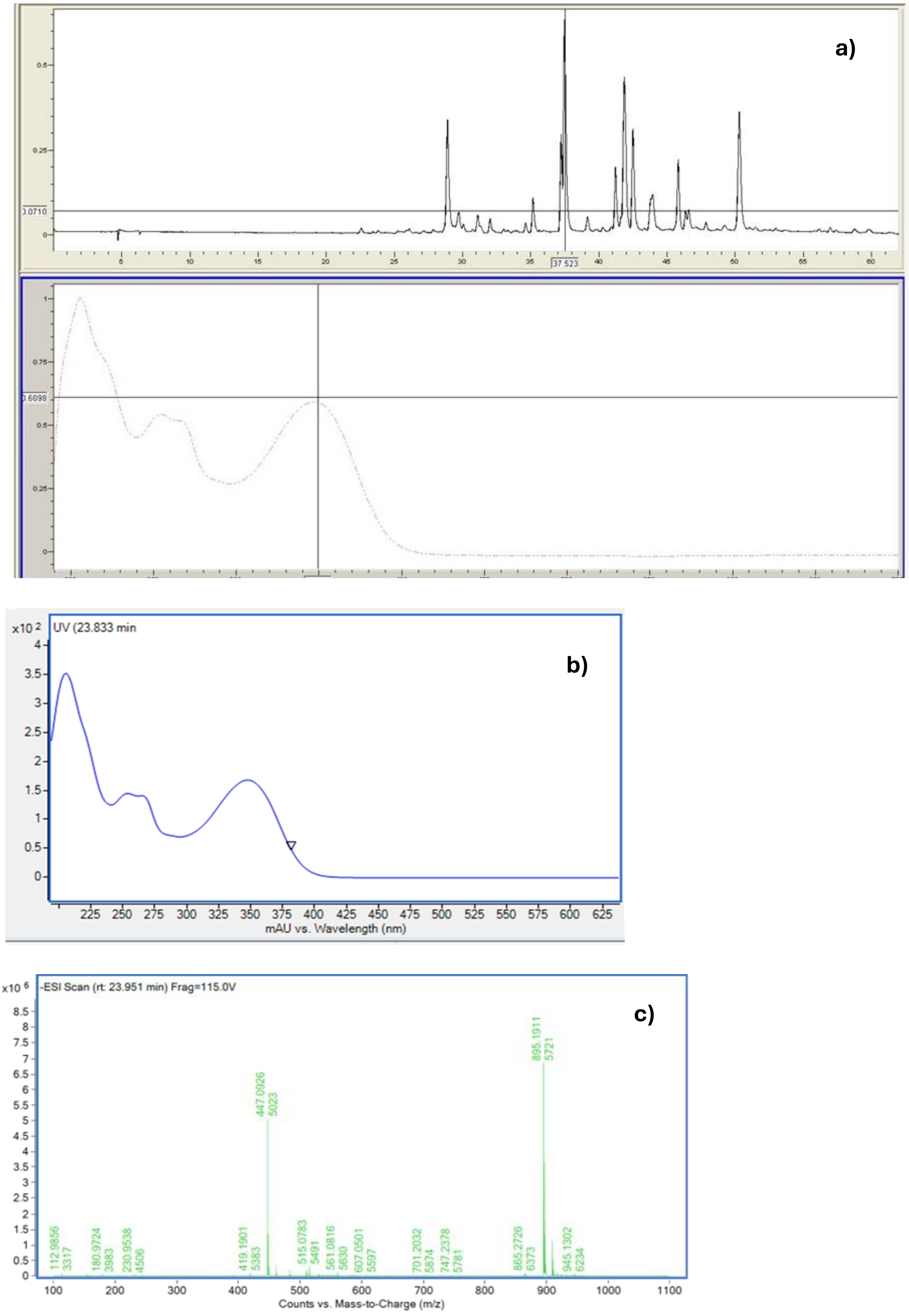

Figure S12. Tentative identification of Peak 10 (HPLC-DAD), Rt = 37.72 min. a) Example chromatogram of a sample with the corresponding UV spectrum acquired by HPLC-DAD. b) UV spectrum of Peak 10 acquired by HPLC-ESI-QTOF. c) HPLC-ESI-QTOF mass spectrum showing the precursor ion at m/z.

Tentative identification of Peak 11 (Apigenin-7-O-Glucoside) : [M-H]=431 m/z

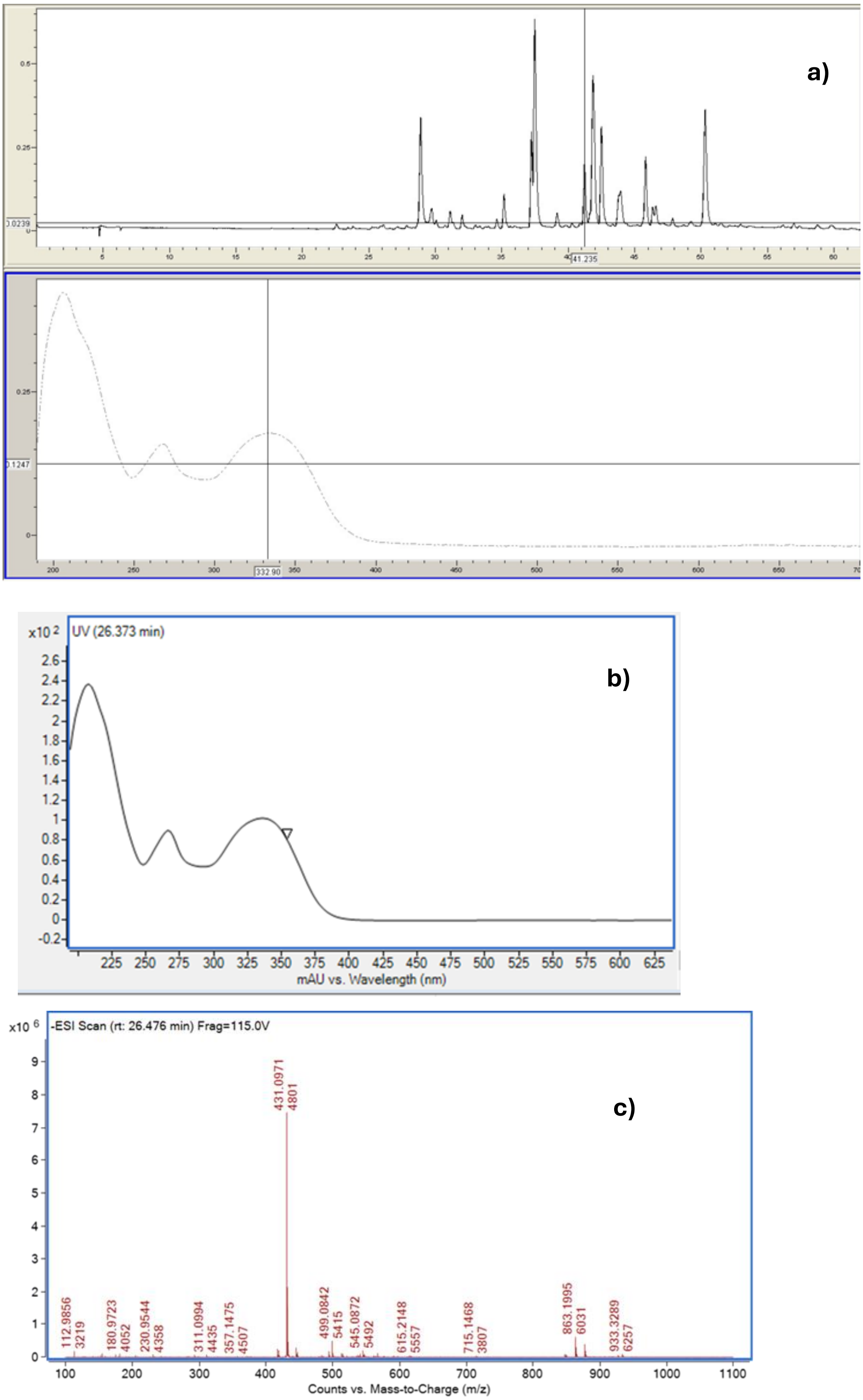

Figure S13. Tentative identification of Peak 11 (HPLC-DAD), Rt = 41.23 min. a) Example chromatogram of a sample with the corresponding UV spectrum acquired by HPLC-DAD. b) UV spectrum of Peak 11 acquired by HPLC-ESI-QTOF. c) HPLC-ESI-QTOF mass spectrum showing the precursor ion at m/z.

Tentative identification of Peak 12 (Apigenin-7-*o*-glucoronide) : [M-H]⁻ = 445 m/z , dime : m/z 891

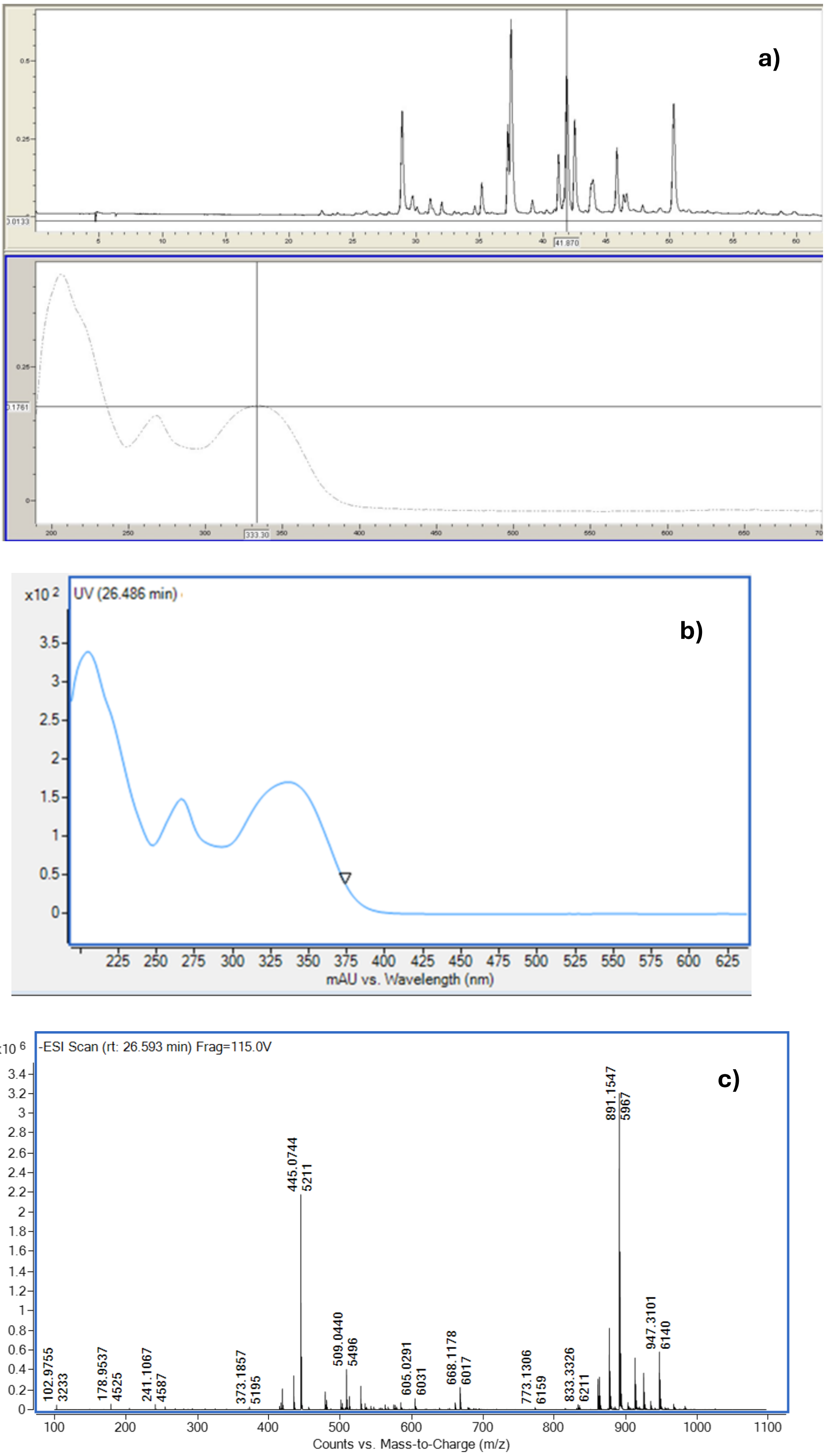

Figure S14. Tentative identification of Peak 12 (HPLC-DAD), Rt = 41.85 min. a) Example chromatogram of a sample with the corresponding UV spectrum acquired by HPLC-DAD. b) UV spectrum of Peak 12 acquired by HPLC-ESI-QTOF. c) HPLC-ESI-QTOF mass spectrum showing the precursor ion at m/z.

Tentative identification of Peak 13 (Methylluteolin-O-glucuronide) : [M-H]= 475 m/z dimer 951 m/z

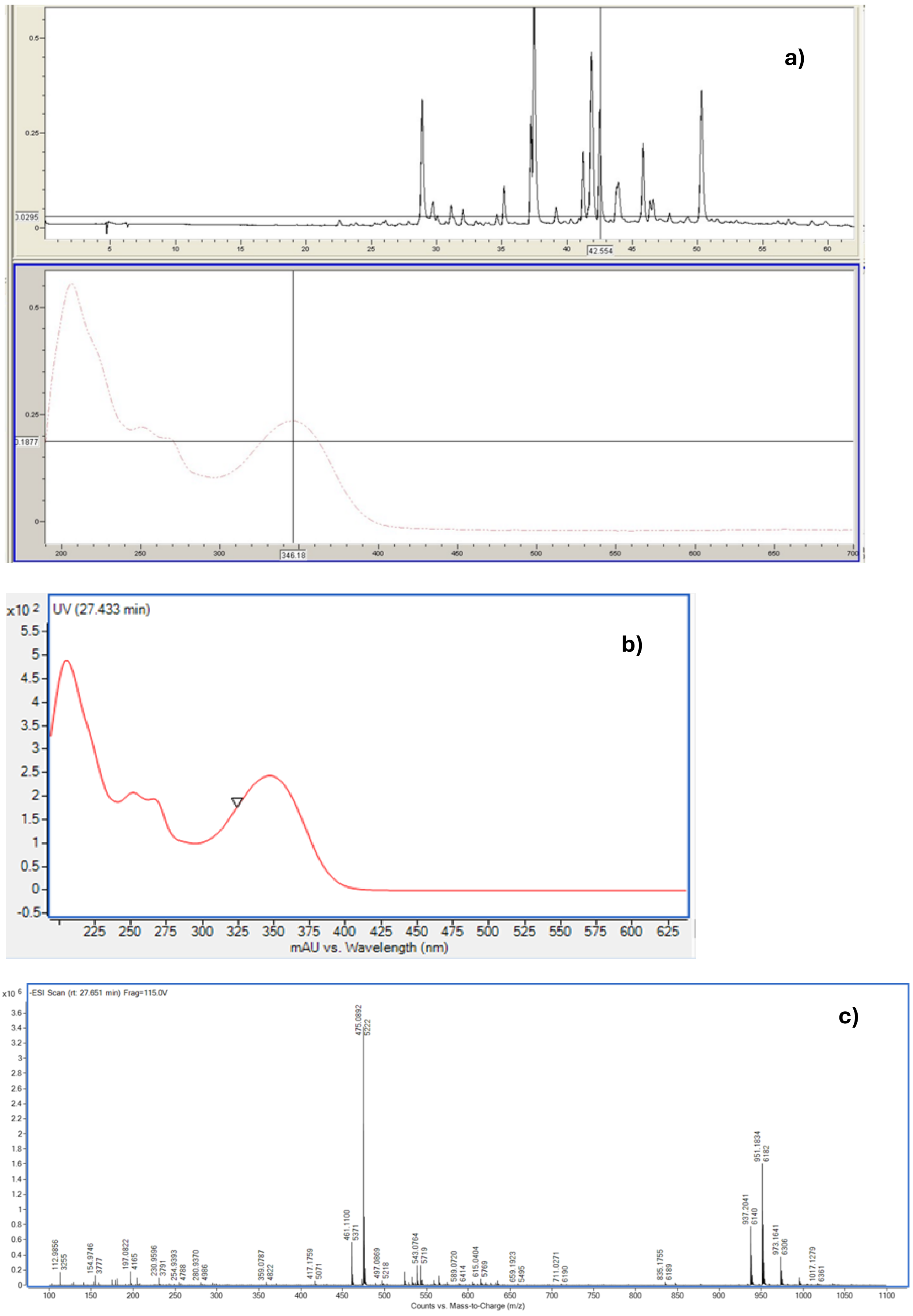

Figure S15. Tentative identification of Peak 13 (HPLC-DAD), Rt = 42.55 min). a) Example chromatogram of a sample with the corresponding UV spectrum acquired by HPLC-DAD. b) UV spectrum of Peak 13 acquired by HPLC-ESI-QTOF. c) HPLC-ESI-QTOF mass spectrum showing the precursor ion at m/z.

Tentative identification of Peak 14 (Rosmarinic acid) $[M-H]^-$ = 359 m/z

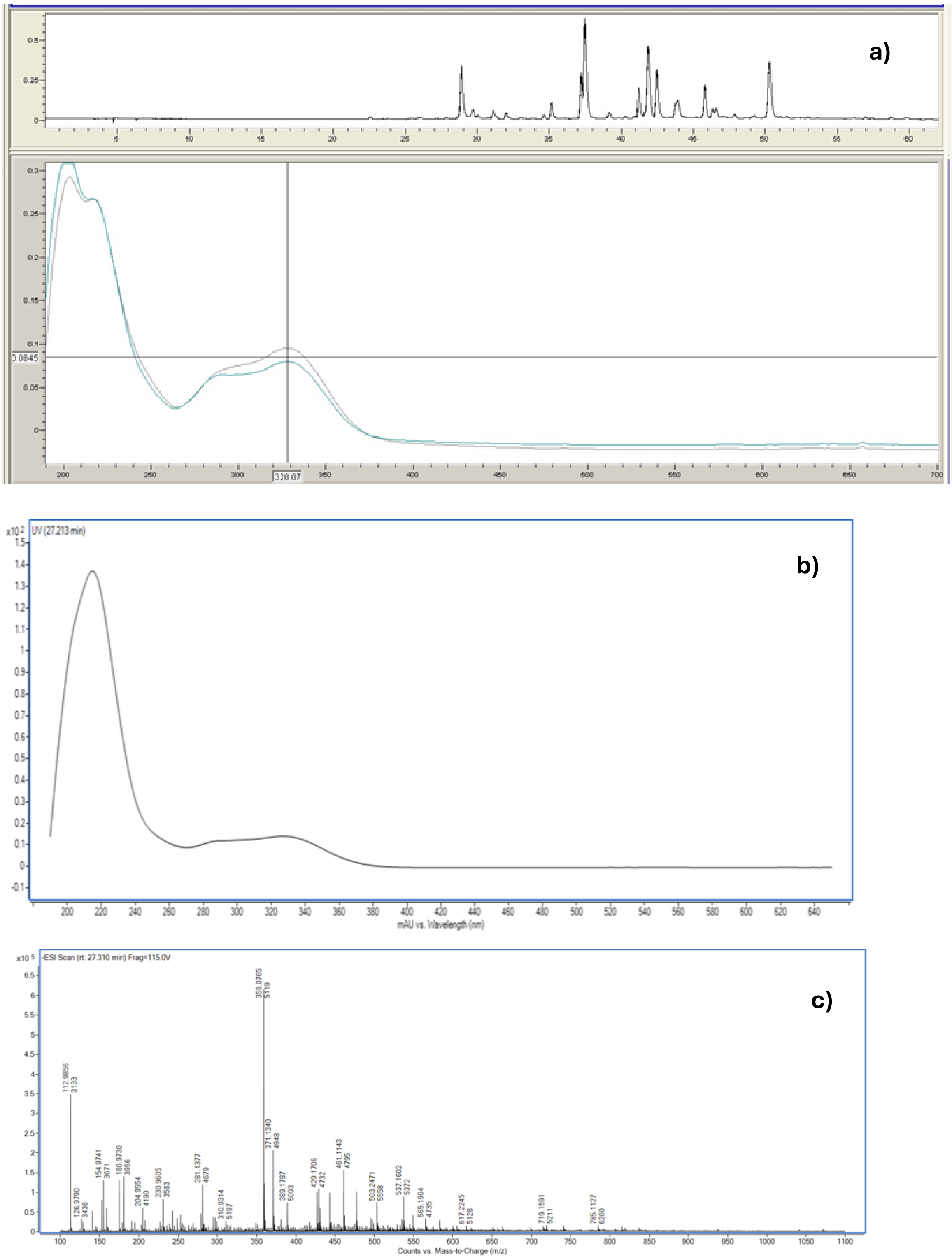

Figure S16. Tentative identification of Peak 14 (HPLC-DAD), Rt = 43.93 min). a) Example chromatogram of a sample with the corresponding UV spectrum acquired by HPLC-DAD. b) UV spectrum of Peak 14 acquired by HPLC-ESI-QTOF. c) HPLC-ESI-QTOF mass spectrum showing the precursor ion at m/z.

Tentative identification of Peak 15 : m/z [M-H]= 473 m/z Apigenin 7-(6''-acetylglucoside), fragment loss of acetyl + glucose (–204 Da) : [M-H-204]$^-$ = m/z 269

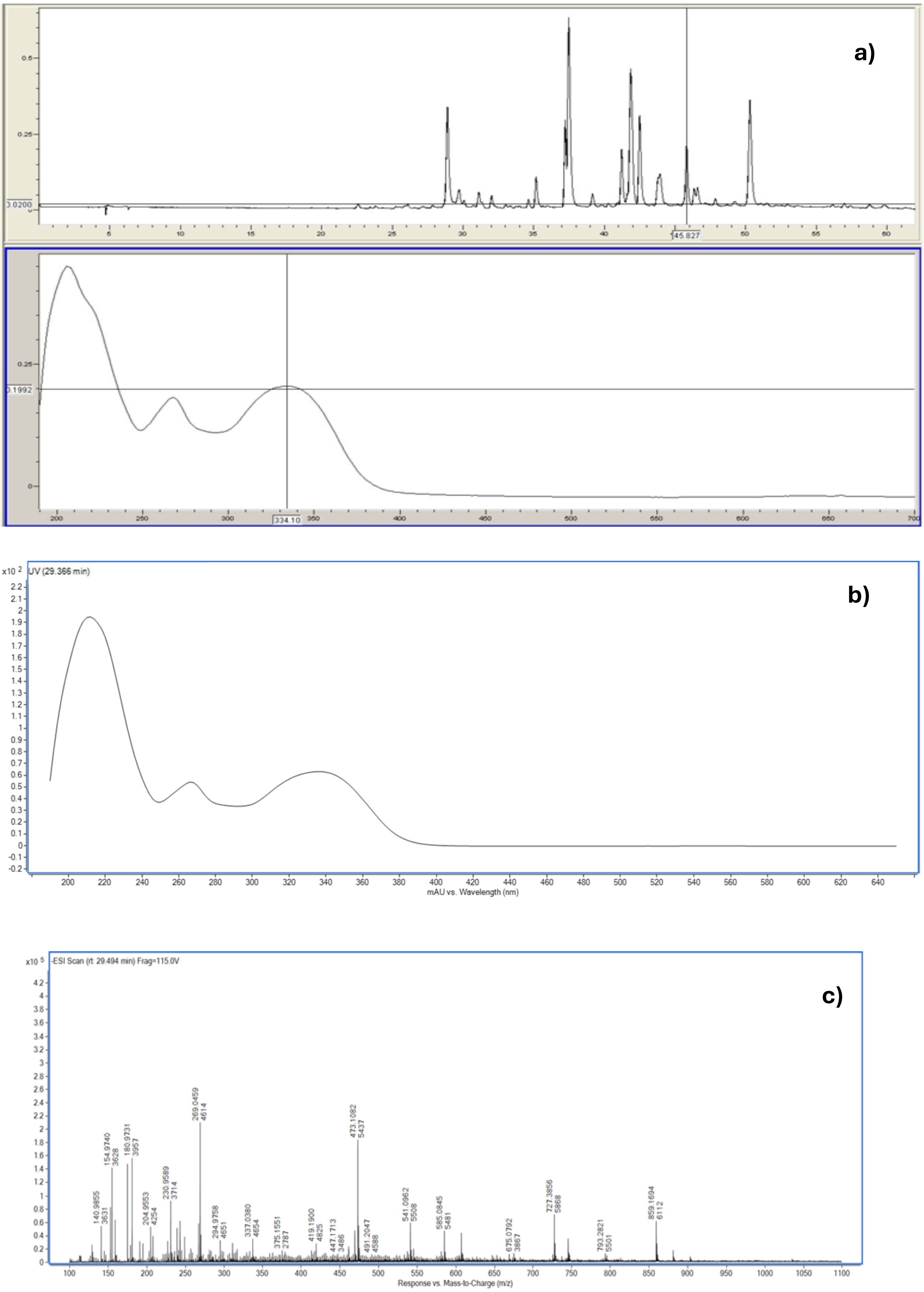

Figure S17. Tentative identification of Peak 15 (HPLC-DAD), Rt = 45.82 min). a) Example chromatogram of a sample with the corresponding UV spectrum acquired by HPLC-DAD. b) UV spectrum of Peak 15 acquired by HPLC-ESI-QTOF. c) HPLC-ESI-QTOF mass spectrum showing the precursor ion at m/z.

Tentative identification of Peak 16 : Salvianolic acid B [M-H]$^-$ = 717 m/z

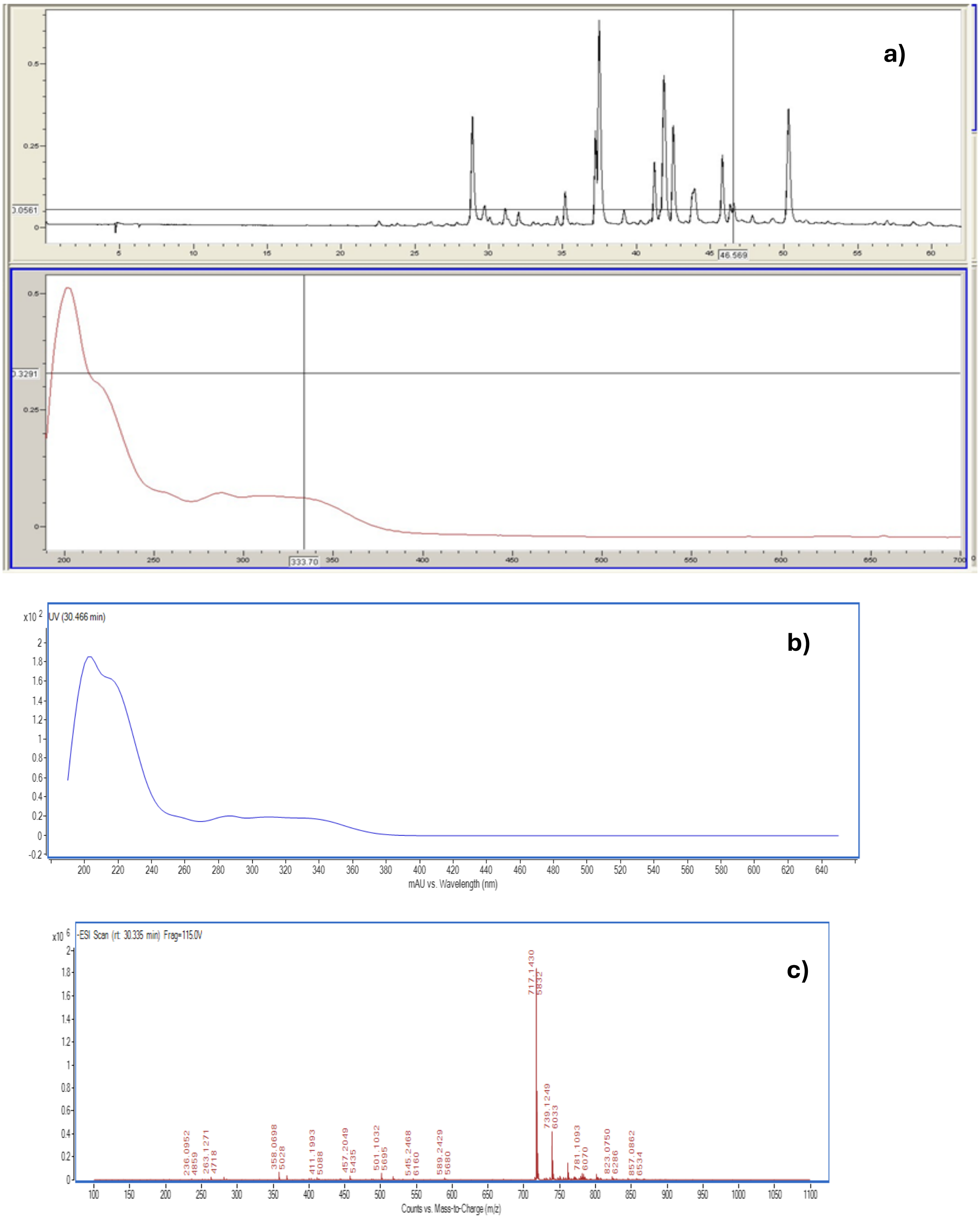

Figure S18. Tentative identification of Peak 16 (HPLC-DAD), Rt = 46.56 min). a) Example chromatogram of a sample with the corresponding UV spectrum acquired by HPLC-DAD. b) UV spectrum of Peak 16 acquired by HPLC-ESI-QTOF. c) HPLC-ESI-QTOF mass spectrum showing the precursor ion at m/z.

Peak 17 UNKNOW1 : [M-H]= 331m/z

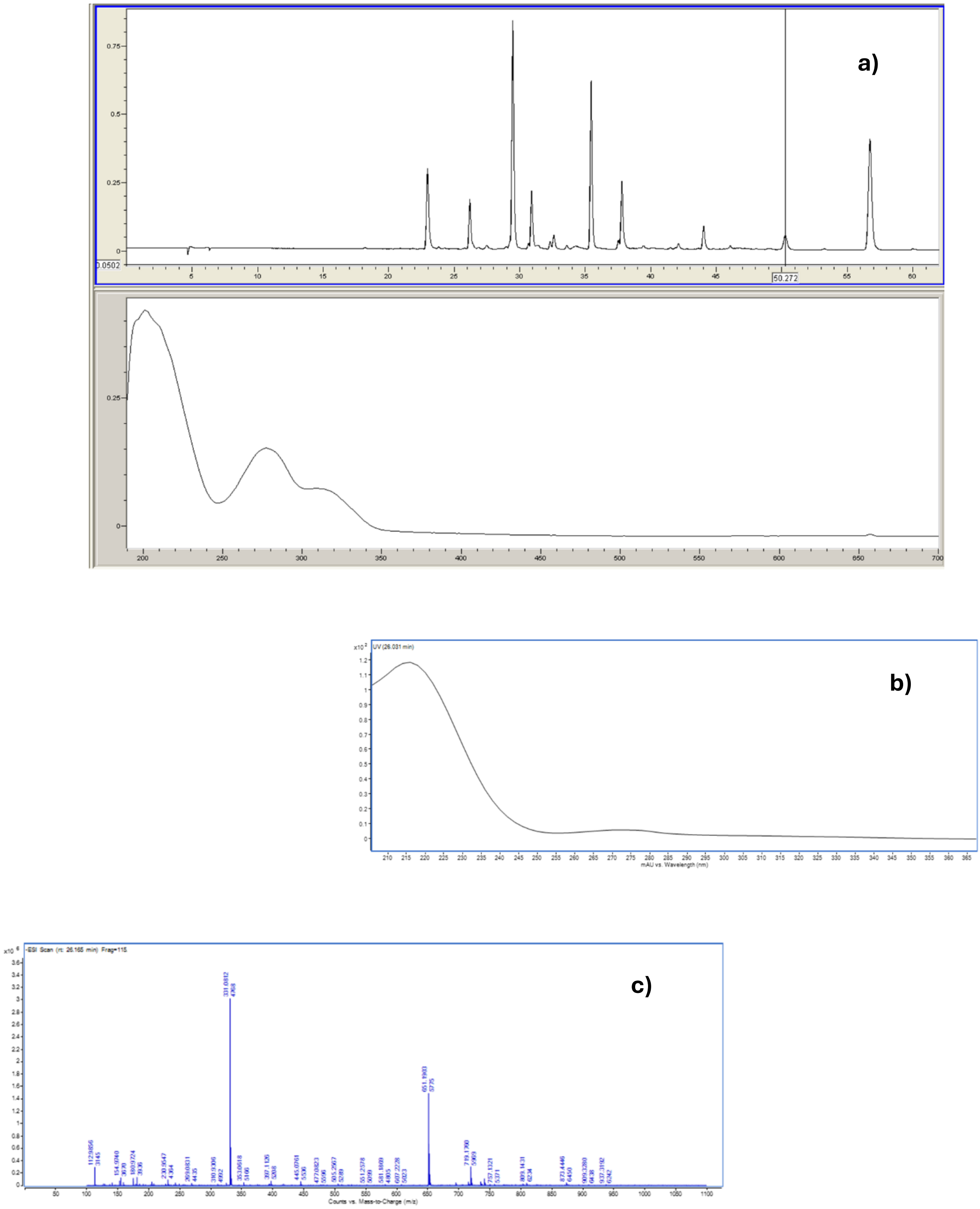

Figure S19. Tentative identification of Peak 17 (HPLC-DAD), Rt = 49.45 min). a) Example chromatogram of a sample with the corresponding UV spectrum acquired by HPLC-DAD. b) UV spectrum of Peak 17 acquired by HPLC-ESI-QTOF. c) HPLC-ESI-QTOF mass spectrum showing the precursor ion at m/z.

Peak 18: UNKNOW 2 m/z [M-H]=501 m/z

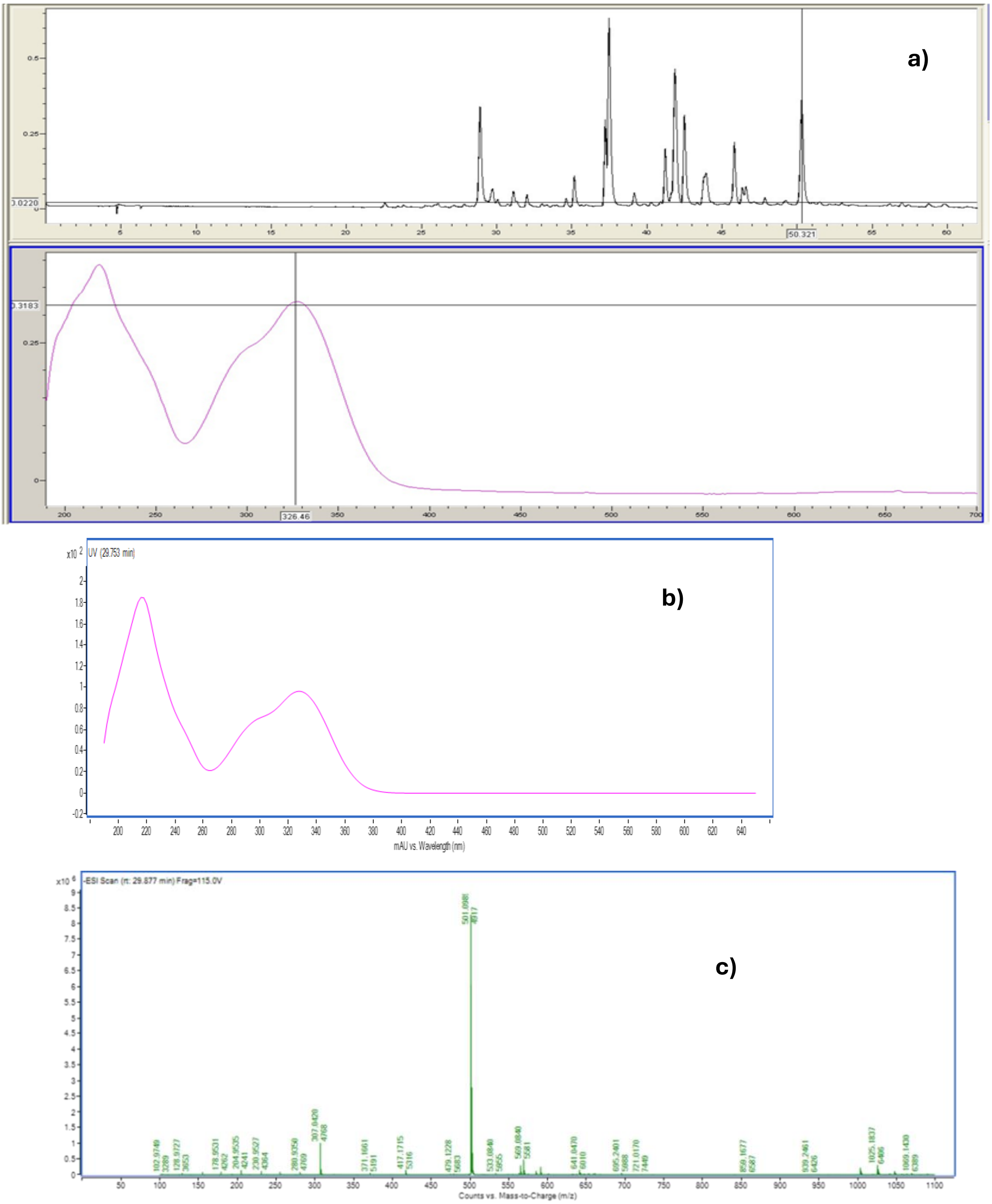

Figure S20. Peak 18 (HPLC-DAD), Rt = 50.28 min). a) Example chromatogram of a sample with the corresponding UV spectrum acquired by HPLC-DAD. b) UV spectrum of Peak 18 acquired by HPLC-ESI-QTOF. c) HPLC-ESI-QTOF mass spectrum showing the precursor ion at m/z.

Peak 19 UNKNOW 3 : m/z [M-H]= 727 m/z

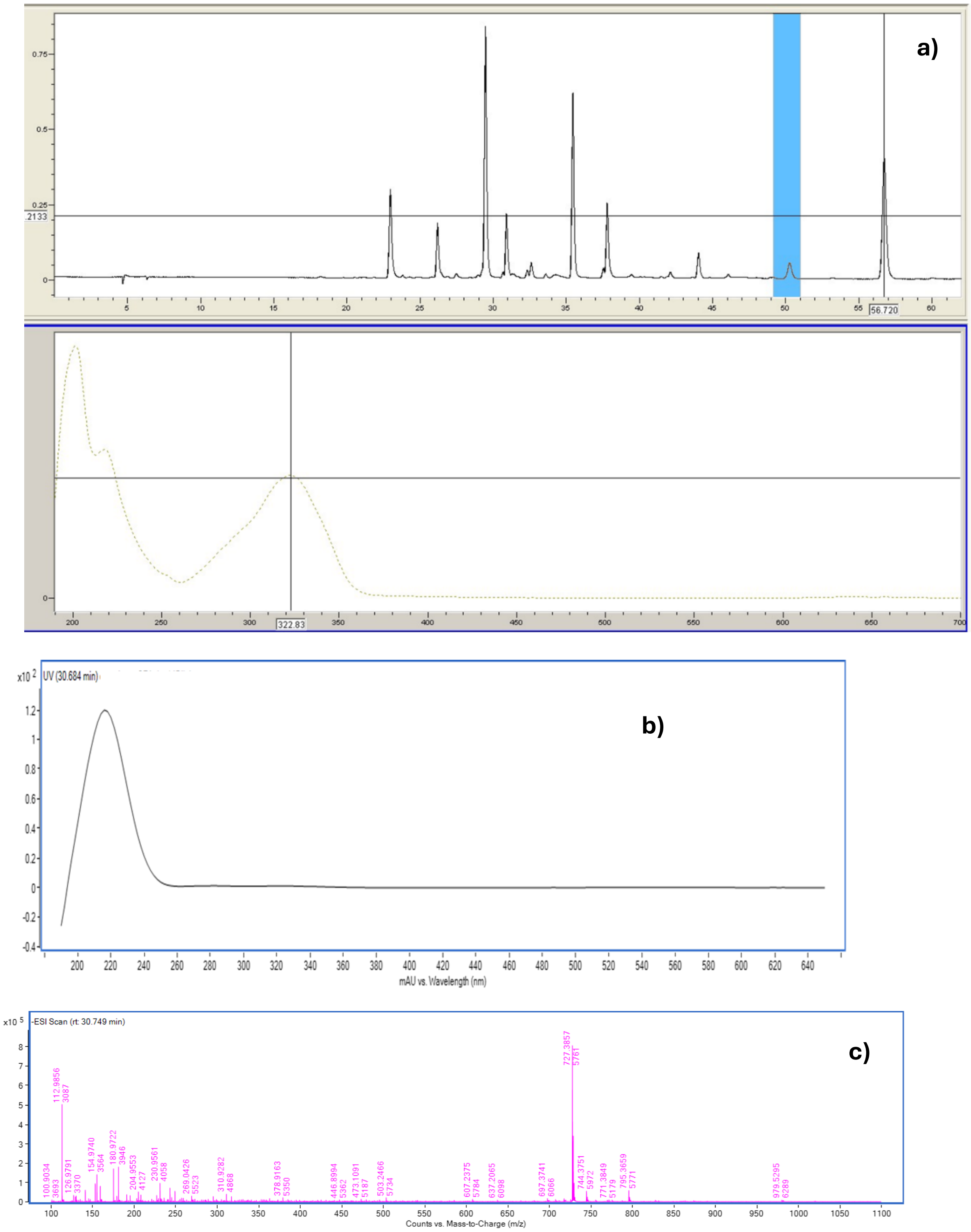

Figure S21. Peak 19 (HPLC-DAD), Rt = 56.72 min). a) Example chromatogram of a sample with the corresponding UV spectrum acquired by HPLC-DAD. b) UV spectrum of Peak 19 acquired by HPLC-ESI-QTOF. c) HPLC-ESI-QTOF mass spectrum showing the precursor ion at m/z.
